# Testing Meningiomas With Methylation Arrays: Insights and Recommendations From a Large Single‐Centre Study

**DOI:** 10.1111/nan.70018

**Published:** 2025-05-13

**Authors:** Fernanda Ruiz, Rossella Rispoli, Zane Jaunmuktane, Ashirwad Merve, Linda D'Antona, Monika Dutt, Felix Sahm, Sebastian Brandner

**Affiliations:** ^1^ Division of Neuropathology, the National Hospital for Neurology and Neurosurgery University College NHS Foundation Trust London UK; ^2^ Department of Neurodegenerative Disease UCL Queen Square Institute of Neurology London UK; ^3^ Department of Clinical and Movement Neurosciences UCL Queen Square Institute of Neurology London UK; ^4^ Department of Histopathology Great Ormond Street Hospital London UK; ^5^ Division of Neurosurgery, the National Hospital for Neurology and Neurosurgery University College Hospitals NHS Foundation Trust London UK; ^6^ Dept. of Neuropathology University Hospital Heidelberg Heidelberg Germany; ^7^ CCU Neuropathology, German Consortium for Translational Cancer Research (DKTK) German Cancer Research Center (DKFZ) Heidelberg Germany

**Keywords:** CNS WHO grading, meningioma, methylation array, model score, prognostication

## Abstract

**Aims:**

Meningiomas are common primary CNS tumours, and their morphological diagnosis is usually straightforward. Their histological grading according to CNS WHO criteria alone provides limited information on recurrence risk. Risk stratification of meningiomas combining WHO grade, methylation class and copy number profile improves prediction of the risk of early recurrence. Because of the frequency of meningiomas in diagnostic practice, applying this prediction algorithm to all meningiomas is financially not viable in most healthcare systems.

**Methods:**

We analysed a retrospective dataset of over 1000 meningiomas from a single centre with methylation arrays to provide guidance on which meningiomas to prioritise for integrated molecular testing and to understand how WHO grades resolve into risk strata.

**Results:**

Approximately 90% of CNS WHO Grade 1 meningiomas were allocated into the methylation class ‘benign’ and also into a low‐risk group. Grade 2 meningiomas were allocated almost equally to either the low‐risk (39%) or intermediate‐risk groups (46%) but occasionally also to the high‐risk group (15%). All grading criteria for CNS WHO Grade 2 meningiomas (brain invasion, mitotic count, cytoarchitectural atypia and histological type) showed a similar risk score distribution as the entire group. Grade 3 meningiomas were allocated to intermediate‐ (26%) or high‐risk groups (74%).

**Conclusion:**

Our data suggest that Grade 2 and 3 meningiomas should be prioritised for methylation profiling. A small proportion of Grade 1 meningiomas may also benefit from integrated molecular analysis, and further research is needed to explore if those histologically benign meningiomas with a predicted increased recurrence risk are associated with distinct demographic or histological characteristics.

## Graphical abstract

Allocation of meningioma grades to risk groups: Risk groups are formed by a combination of meningioma WHO grade, methylation class and patterns of chromosomal losses. Most (89%) Grade 1 meningiomas were allocated to the low‐risk group, and the majority (74%) of Grade 3 meningiomas to the high‐risk group. Instead, *G*rade 2 meningiomas show an allocation into low‐ (39%), intermediate‐ (46%) and high‐risk (15%) groups. Further analysis of the *G*rade 2 criteria (brain invasion, mitotic count and histological type) shows no strong preference for brain invasion or mitosis for the low‐, intermediate‐ or high‐risk group. The study concludes that Grade 2 meningiomas benefit most from methylation profiling with risk stratification.

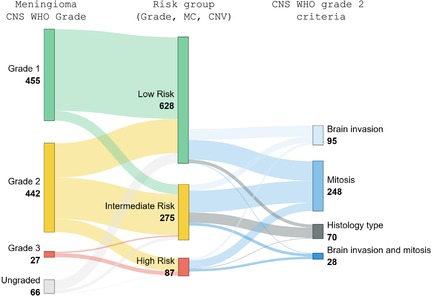

**Summary**

- Approximately 90% of CNS WHO Grade 1 meningiomas are allocated to the methylation class meningioma, benign, and have an integrated score corresponding to a low risk of recurrence.
- CNS WHO Grade 2 and 3 meningiomas benefit most from methylation profiling. Meningiomas that are CNS WHO Grade 2 due to brain invasion, mitotic count or histological type each have a relatively similar allocation to methylation classes, benign (average 65%), intermediate (average 30%) or malignant (average 4%).
- One‐third of histologically benign CNS WHO Grade 1 meningiomas and half of CNS WHO Grade 2 meningiomas have chromosome 1p and 22q loss.

## 1 Introduction

Meningiomas are the most common primary CNS neoplasms in adults, with an age‐adjusted rate of nearly 10 cases per 100,000 population (40.8% of CNS tumours) [1]. The histological diagnosis and WHO grading of meningiomas follow well‐established criteria [2] set out in the WHO classification for CNS tumours. The current CNS WHO classification identifies 15 histological subtypes and three grades [3]. CNS WHO Grade 1 (henceforth, all ‘CNS WHO Grades’ are simplified as ‘grades’ in the main text) meningiomas are histologically bland; in most instances are of the meningothelial, fibrous, transitional and psammomatous subtypes; and have 3 or less mitotic figures per 10 high‐power fields (HPF; 0.16 mm^2^). Meningiomas correspond to Grade 2 (atypical) if they have one or more of the following criteria: (i) a mitotic count of 4–19 in 10 HPF (1HPF = 0.16 mm^2^), i.e., equivalent to ≥ 2.5 mitoses/mm^2^; (ii) brain invasion; (iii) are of the histological subtypes chordoid or clear cell [4]; or (iv) three or more of the histological features (hypercellularity, sheet‐like growth, prominent nucleoli, spotty necrosis or small cell change); or a combination of more than one of the above criteria. Virtually, all clear cell meningiomas carry *SMARCE1* mutations [5]. Grade 3 (anaplastic/malignant) meningiomas typically have a high‐grade appearance but are graded accordingly when they have 20 or more mitotic figures in 10 HPF (0.16 mm^2^) or ≥ 12.5 mitoses/mm^2^ [6]. Prior to the revised CNS WHO 2021 criteria, the rhabdoid and papillary histological subtypes were also considered to correspond to CNS WHO Grade 3. The CNS WHO 2021 classification recommends that for rhabdoid or papillary histological subtypes, the grade should be assigned by applying the criteria for Grade 2 atypical or Grade 3 anaplastic meningioma and not be based on rhabdoid or papillary histology alone [7], whereas Grade 1 is not an option. In addition, the presence of *CDKN2A/B* gene deletion or a *TERT* promoter mutation was added as a criterion for Grade 3 meningioma in the 2021 CNS WHO classification [3]. Although the histological grading system provides some degree of prognostication, with mitotic figures being the most reproducible [8], it is limited by its reliance on other histological features, some of which can be subjective. The prognostication of risk of early recurrence for meningiomas was significantly improved by establishing methylation profiles that captured clinically more homogeneous groups and had a higher power for predicting tumour recurrence than the WHO classification [9]. Subsequently, further efforts were made to refine prognostication by adding further layers of molecular stratification, such as transcriptional profiles [10, 11] or chromosome copy number changes [12, 13]. The approach, which combines histological grade, chromosome copy number profile, methylation profile [12, 13] and calculation of a model score, is well‐suited for integration into the molecular diagnostic workflow [4, 14]. The model score is calculated from WHO grade, Methylation Family (MF) and chromosomal copy number, as follows: WHO Grade 1 = Score 0, Grade 2 = Score 1, Grade 3 = Score 2; MF benign = Score 0, MF intermediate = Score 2 and MF malignant = Score 4; and loss of chromosomes 1p, 6q or 14q, with a score of 0 when no loss is present, score of 2 when 1 or 2 of these chromosomes is lost and score of 3, when all three chromosomal arms are lost. Scores of each category are added to form an integrated model score. Model scores of 0, 1 or 2 are considered ‘low risk’; scores of 3, 4 or 5 correspond to ‘intermediate risk’; and scores from 6 to 9 are ‘high risk’. The limitation of the histological (WHO) grading scheme, with a review of grading criteria and recommendation of additional molecular tests, is also reflected in the recent cIMPACT‐NOW Update 8: clarifications on molecular risk parameters and recommendations for WHO grading of meningiomas [15].

Because meningiomas are the most common brain tumours in adults, they represent a comparably high diagnostic volume in neuropathology departments. Considering the cost of sample workup for methylation array analysis and reporting of results, testing of all meningiomas is financially unsustainable in the majority of neuropathology departments in most healthcare systems. Consequently, it is essential to determine which meningiomas would benefit most from additional analysis using methylation array technology. Therefore, we analysed a cohort of over 1000 meningiomas from our clinical practice, integrating CNS WHO histological grade, methylation class (MC) and subtypes and copy number profile using validated, published methodology [9, 12, 13].

## 2 Methods

### 2.1 Patient Cohort and Clinical Workflow

**FIGURE 1:**
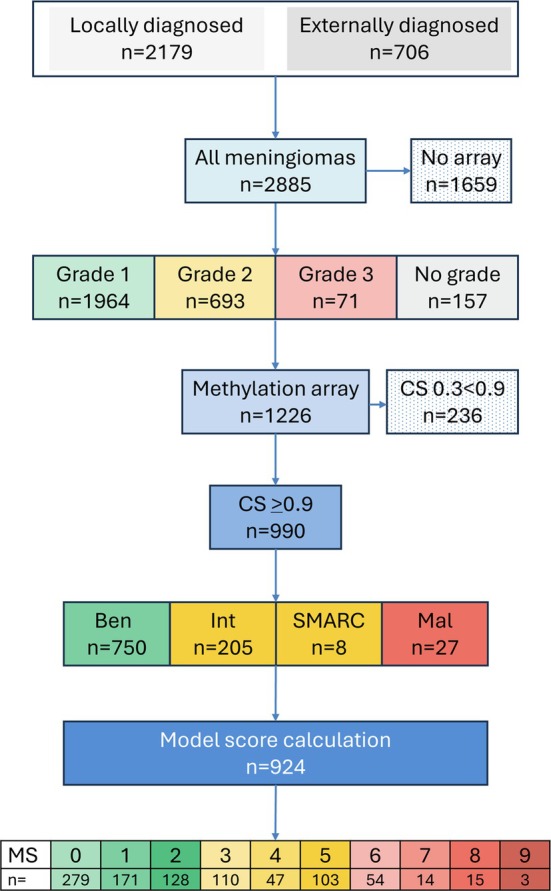
Diagnostic workflow in this study. From all locally diagnosed and externally diagnosed meningiomas (*n* = 2885), a subset (*n* = 1226) underwent methylation array analysis. Arrays with a calibrated score of 0.9 and higher (*n* = 990) were used to establish methylation classes. For the calculation of the model score (which requires the WHO grade), the subset of graded meningiomas (*n* = 924) was included.

The Division of Neuropathology at Queen Square has two referral pathways. The primary diagnostic pathway comprises samples that are referred from several neurosurgical centres (including our hospital) for histological diagnosis and molecular pathology workup (‘locally diagnosed’). These samples are examined by a team of neuropathologists based in the Division of Neuropathology, Queen Square, London. The decision to test samples further is made by the Queen Square neuropathologists, either based on the histological impression or following discussion in the multidisciplinary team meetings (MDTM or ‘tumour board’). In addition, the Division of Neuropathology receives referrals specifically for molecular testing of tumours for which the initial histological diagnosis and the decision to perform further molecular tests is made by external neuropathologists (‘externally diagnosed’) (Figure 1).

### 2.2 Tissue Samples and Methylation Array Analysis

Sections of the formalin‐fixed paraffin‐embedded (FFPE) samples selected for methylation array analysis were mounted on glass slides (10 μm thick, 8 consecutive slides). On an adjacent H&E‐stained section (4 μm), a suitable tumour area was identified to maximise the inclusion of viable tumour‐containing tissue. A tumour content of at least 80% was selected where possible, and nonneoplastic tissue (e.g., dura mater) and blood were excluded, if present. Slides with mounted tissue were dewaxed and air‐dried. The tissue selected for the analysis was scraped off and collected in lysis buffer, and DNA was extracted with the Maxwell FFPE DNA Purification Kit on a Maxwell 16 extractor. The DNA extraction procedure was carried out according to manual #TM349 for DNA extraction (Promega). DNA was quantified, and A260/A280 ratios were determined on a Nanodrop 8000 Spectrophotometer (ThermoFisher). An A260/A280 ratio of ~1.8 was considered to represent sufficient purity to proceed with the methylation study. Based on the DNA quantification steps as determined previously, we aim at an input of 250 ng as a minimum and ideally at 500 ng of DNA from each sample for bisulphite conversion. The EZ DNA Methylation Kit (Zymo D5024) was used for DNA bisulphite conversion. All steps were performed according to the manufacturer's guidelines. The EPIC (850 k) methylation array v1 was used to obtain genome‐wide DNA methylation profiles for FFPE tumour samples, according to the manufacturer's instructions (Illumina). DNA methylation arrays were processed at the UCL Genomics facility at the UCL GOS Institute of Child Health. On‐chip quality metrics of all samples were carefully controlled. Methylation analysis data (idat files) were transferred to the Division of Neuropathology and processed on a local copy of the Classifier (www.molecularneuropathology.org). Initially (in 2018), meningiomas were processed using the meningioma classifier 2.4 [13]. In 2023, following the publication of the equivalency study [12], the brain classifier 12.6 (the locally installed version of the 12.5 web version) was used. To obtain homogeneous data, all samples were reprocessed with the DKFZ 12.6 classifier version.

### 2.3 Data Analysis

An export of the datasets was performed from the laboratory information management system, returning the histology ID, demographic data, medical record number (MRN), diagnosis field and the microscopic description field. The data were cross‐referenced with a separate table containing the sentrix ID (a unique identifier assigned to each Illumina microarray chip combined with the sample position on the chip) and the histology ID. Tumour recurrences were identified by highlighting duplicate medical record numbers.

Histological features relevant for tumour grading (brain invasion, mitotic count, histological type [such as chordoid or clear cell], cellularity, presence of nucleoli, small cell change, patternless sheet‐like growth and spotty necrosis) were determined by a readout from the microscopic description data field or the diagnostic comment field. When assessing referred cases, we occasionally found a discrepancy (both ways) between the mitotic count determined by the referring pathologist's report and the mitotic count on the section analysed by us. In such instances, the higher value was used for grading and thus contributed to the model score. In rare instances, the referred diagnosis of a papillary or rhabdoid meningioma was revised upon review.

All Infinium Methylation EPIC 850K array data (idat files) were reprocessed with the DKFZ classifier 12.6 and the conumme R package to export chromosomal copy number data (‘conumee’ package for R; https://github.com/dstichel/conumee/). CNV data are then processed to map segments to chromosomal arm annotations and to determine cumulative gains and losses within defined genomic regions relevant for the risk stratification, i.e., chromosome 1p, 6q and 14q. The log2 ratio between test sample and reference values was used to calculate relative abundance (i.e., CNV) across regions, and this is interpreted as copy number difference. References were derived from a normalised methylation dataset (Mset), using the MNPpreprocessIllumina function in mnp.v12epicv2. The MNPcnv function was used to detect CNVs, and it loads a predefined reference dataset (refF_epic) and annotations and then runs the CNV calling using conumee2.0::CNV.fit. A log2 ratio of −0.1 indicates the loss of one copy. Next, the proportion of chromosomal arm loss was calculated, whereby loss of more than 5% of a chromosomal arm qualified for the scoring as ‘loss’ [12, 16, 17]. Homozygous deletion of *CDKN2A/B* was indicated by a log2 value below 0.4.

## 3 Results

### 3.1 Diagnostic Pathways and Samples Included in the Study

**FIGURE 2:**
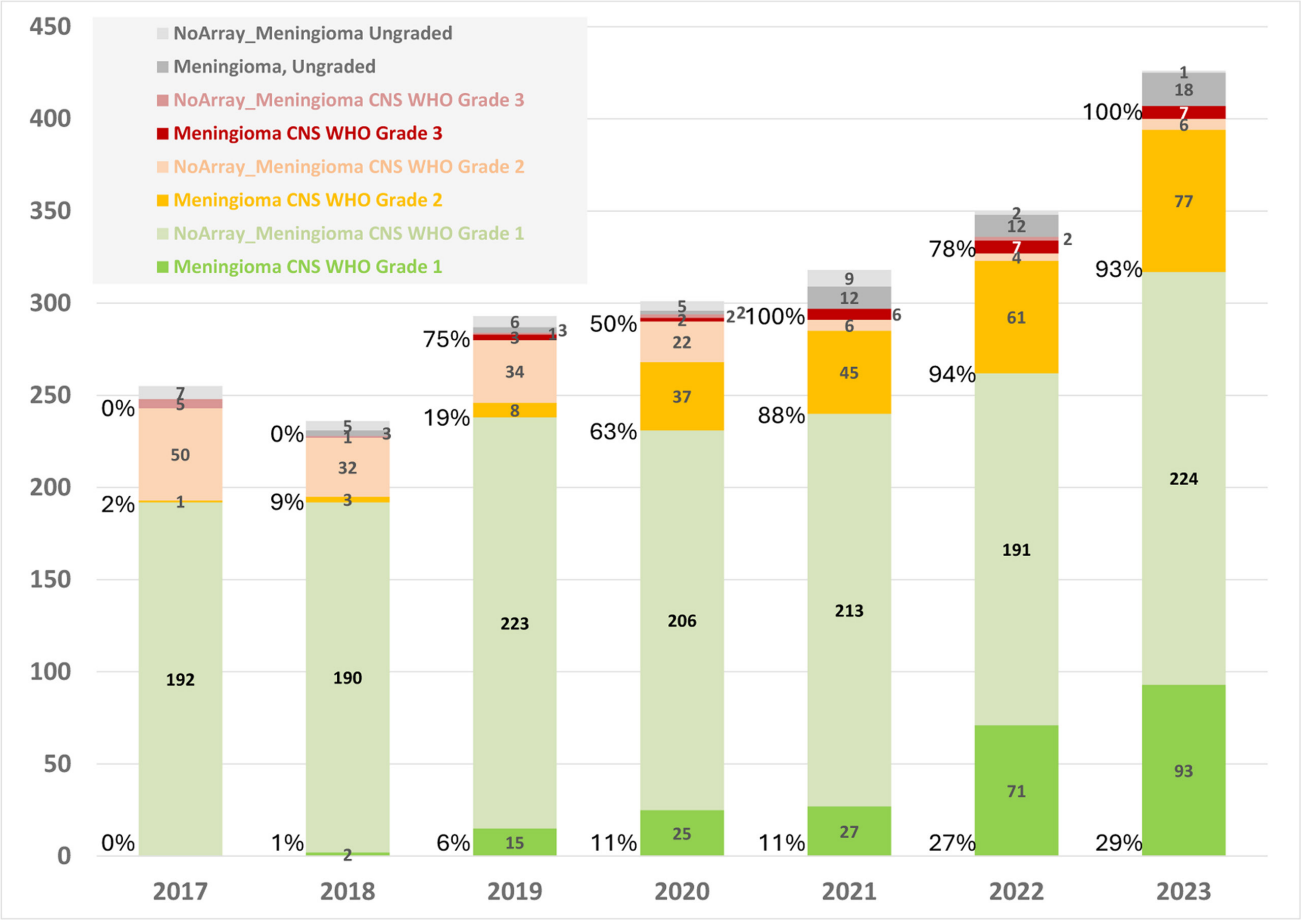
Methylation profiling of meningiomas in the standard diagnostic pathway: 2179 meningiomas were diagnosed between January 2017 and December 2023. The graph indicates the proportion of Grade 1, 2 and 3 meningiomas, as well as ungraded meningiomas. There is a continuous increase of methylation arrays for risk stratification of meningiomas. 29% of Grade 1, 93% of Grade 2 and 100% of Grade 3 meningiomas were profiled in 2023. See also Table S1 for corresponding data.

Between January 2017 and December 2023, 2885 meningiomas (patients aged 16–91) were diagnosed in the Division of Neuropathology. Of these, 2179 meningiomas were diagnosed as part of the standard diagnostic pathway, i.e., a primary (initial) diagnosis was established in the Division of Neuropathology (Figures 1 and 2 and Table S1) (‘Locally diagnosed’). The 2179 locally diagnosed cases consisted of 80.1% Grade 1 meningiomas, 18.3% Grade 2 and 1.5% Grade 3 tumours, with malignant forms being more prevalent in males (1.4%) than in females (0.6%) [1].

Seven hundred six meningiomas were diagnosed elsewhere and were referred (‘Externally diagnosed’), and of these, 686 (97.2%) had been referred for methylation array profiling, in particular for the stratification of early recurrence risk (Table S2 and Figure S1). In this study, 1226 meningioma methylation profiles were included. Figure 2 and Table S1 show the frequency of methylation array profiling of meningiomas in the diagnostic pathway of locally diagnosed cases, i.e., as a result of the diagnostic decision of the neuropathologist. The use of methylation arrays for the prognostication of recurrence has continuously increased between 2017 and 2023. In 2023, 29% of Grade 1 meningiomas, 93% of Grade 2 meningiomas and 100% of Grade 3 meningiomas were analysed with methylation arrays. A proportion of meningiomas (19/426, 4.2%) could not be graded morphologically, either due to insufficient sample size or, more frequently, due to mechanical or cauterisation artefacts obscuring diagnostic features relevant for histological grading. Tables S1 and 2 show that in 2023, 128 of 132 (97%) Grade 1 meningiomas, 191/191 (100%) of Grade 2 and 15/15 (100%) of Grade 3 meningiomas were externally diagnosed (Figure 1) and referred for methylation profiling and integrated reporting. Meningiomas were referred at noticeable numbers after 2019, following the publication of the meningioma classifier in 2017 [9], and a further significant increase was the subsequent publication of the integrated risk score (‘model score’, also referred to as ‘integrated score’), which further facilitated a technically simple, logical, reproducible and evidence‐based reporting, using an established and technically validated method [13].

### 3.2 Allocation of Meningioma Grades to MCs

**FIGURE 3:**
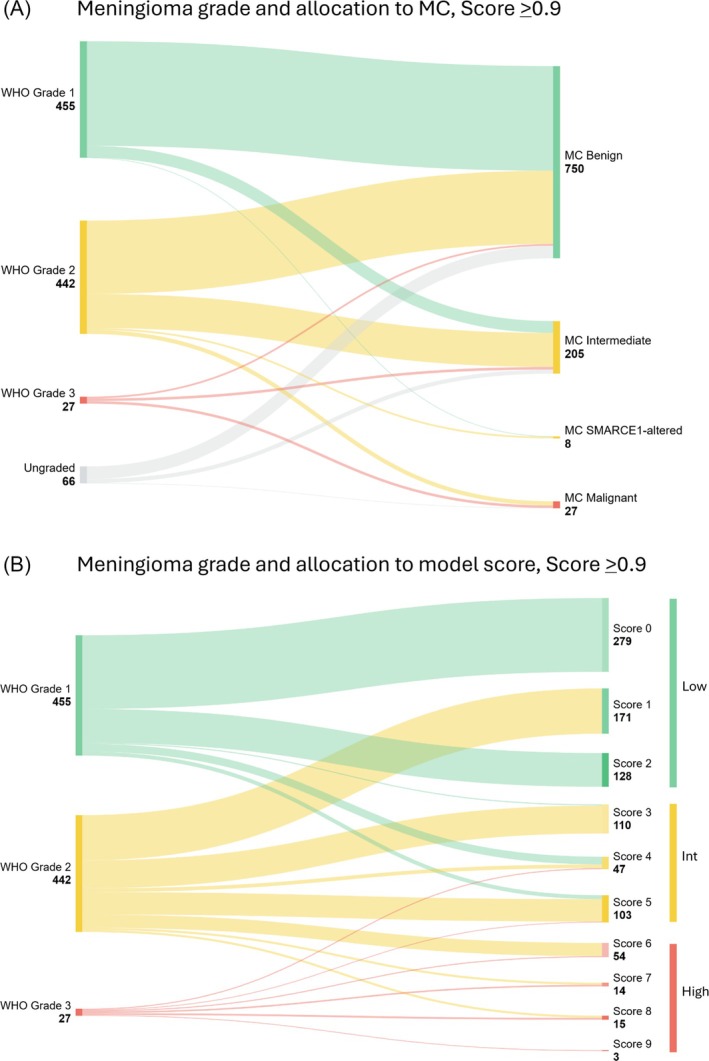
(A) Outcome of the methylation profiling of all referred and internally diagnosed meningiomas, with a calibrated score of ≥ 0.9. A majority of Grade 1 tumours are allocated to the methylation class benign, whereas Grade 2 tumours allocate approximately 60% to the MC benign and the remaining tumours into MC of intermediate and rarely malignant. See also Table S3 for corresponding data. (B) Outcome of the methylation profiling of all referred and internally diagnosed meningiomas after calculation of the model score. Of the Grade 1 meningiomas, a significant majority allocate to these three low scores of 0, 1 and 2. Instead, Grade 2 meningiomas show a wider distribution across many model scores courses, underpinning the importance of prioritising these tumours for methylation profiling with risk prediction.

We first examined how meningiomas of Grades 1, 2 and 3 and ungraded meningiomas allocate to the predicted MCs meningioma benign, intermediate, malignant and the MC meningioma, *SMARCE1*‐altered, and how these meningiomas resolve into the model scores of 0–9. To this end, we first stratified the output from the classifier version 12.6 into meningiomas with a calibrated score of ≥ 0.9 (*n* = 990) (Table S3 and Figure 2) and meningiomas with calibrated scores of 0.3 < 0.9 (*n* = 237) (Table S4 and Figure S2). The latter were excluded from the subsequent detailed analysis to ensure that the analysis was based on robust scores. Figure 3A (corresponding data in Table S3, bottom part) shows that of the 455 Grade 1 meningiomas, 408 (89.7%) were allocated to the MC benign, and the remaining Grade 1 meningiomas were allocated to the methylation subtype intermediate (*n* = 46, 10.1%) and a single case to the MC meningioma, clear cell subtype, *SMARCE1*‐altered. Meningiomas graded as Grade 2 (incorporating grading based on brain invasion, mitotic count, histological characteristics or a combination of brain invasion and mitotic count) show a wider range, with two‐thirds (286/438, 65%) classifying as benign, and approximately one‐third (133/442, 30%) classifying as intermediate, and a small number into the MC *SMARCE1*‐altered (7) or the MC malignant (16/442, 3.7%). Meningiomas that histologically corresponded to Grade 3 (*n* = 27, 2.7% of all tumours classified with a score ≥ 0.9) were distributed relatively evenly into the three MCs, namely, benign (7/27, 26%), intermediate (10/27, 37%) or malignant (10/27, 37%). Finally, in the locally diagnosed cohort, we had 85/2179 (3.9%) ungraded meningiomas (e.g., due to a small sample size or mechanical artefact preventing from assessing cytoarchitecture and mitotic activity), of which 50 underwent methylation profiling. The remaining 35 ungraded meningiomas were unsuitable for analysis due to insufficient or poor‐quality DNA. Of the additional 69 referred ungraded meningiomas, 66 underwent profiling, i.e., 116 ungraded meningiomas were profiled. Sixty‐six of these 116 tumours returned a calibrated score of 0.9 and higher,17 returned a calibrated score between 0.3 and 0.9 (Table S4), and the remaining tumours did not classify with a calibrated score of up to 0.3. The 66 ungraded tumours with CS ≥ 0.9 were included in further detailed evaluation. The majority (49/66, 74%) were classified as ‘benign’, 16/66 (24%) were classified as ‘intermediate’ and 1/66 (1.5%) was classified as ‘malignant’. None of the ungraded meningiomas were classified as *SMARCE1*‐altered. The allocation of MCs of ungraded meningiomas corresponds approximately to the overall frequency of allocation to the MCs, where 49/66 (74%) of ungraded meningiomas, and 750/990 (76%) of all meningiomas were classified as ‘benign’, 16/66 (24%) of ungraded meningiomas compared with 205/990 (21%) of all meningiomas were classified as ‘intermediate’, and 1/66 (1.4%) compared with 27/990 (2.7%) of all meningiomas were classified as ‘malignant’. Therefore, profiling all meningiomas that cannot be graded histologically, such as those with limited size or poor morphological preservation, can be useful for risk of recurrence stratification purposes. Ungraded meningiomas comprise 66/990 (6.7%) in the high‐scoring group (CS 0.9–1.0; Table S3) and 17/237 (7.2%) in the low‐scoring group (0.3 < 0.9; Table S4) and are therefore similarly represented in both cohorts of higher and lower calibrated scores, indicating that the reasons for difficulty in histological grading do not affect the calibrated scores.

### 3.3 Allocation of Meningiomas of CNS WHO Grades 1, 2 and 3 Into Model (Risk) Scores

The combination of histological grade, MC and copy number profile allows the establishment of a model score to predict the risk of early recurrence [13]. Figure 3B illustrates how meningiomas diagnosed as Grades 1, 2 or 3 resolve into the model scores of 0–9 (data in Table S5). As the calculation of the model score requires a WHO grade, we have omitted ungraded meningiomas and included 924 remaining samples in this analysis. The majority of Grade 1 meningiomas (*n* = 455) scored 0 or 2, i.e., corresponded to the low‐risk group (*n* = 407, 89%). Only 11% allocated to scores of 3–5, i.e., corresponding to the intermediate risk of early recurrence group. None of the Grade 1 meningiomas were allocated to the high‐risk groups (i.e., scores of 6–9). Meningiomas that histologically corresponded to Grade 2 (*n* = 442) were allocated predominantly to the low‐risk groups (*n* = 171, 39%) or the intermediate‐risk groups (*n* = 205, 46%) and to a lesser extent to the high‐risk groups (*n* = 66, 15%) (Table S5 and Figure 3B). Grade 3, anaplastic meningiomas (*n* = 27) were never allocated to the low‐risk group but instead were allocated predominantly to the high‐risk groups 6–9 (*n* = 20, 74%) and occasionally to the intermediate‐risk Groups 4 and 5 (*n* = 7, 26%).

### 3.4 Allocation of CNS WHO Grade 2 Meningioma to MCs

Meningiomas are graded as Grade 2 if they meet one or more of the following criteria, (i) mitotic activity (4–19 per 10 high power fields), (ii) brain invasion, (iii) are of chordoid or clear cell histological type or (iv) show at least 3 of 5 other features of cytoarchitectural atypia, such as high cellularity, small cell change with a high nucleus to cytoplasm ratio, prominent nucleoli, sheet‐like growth and foci of spontaneous tumour necrosis.

**FIGURE 4:**
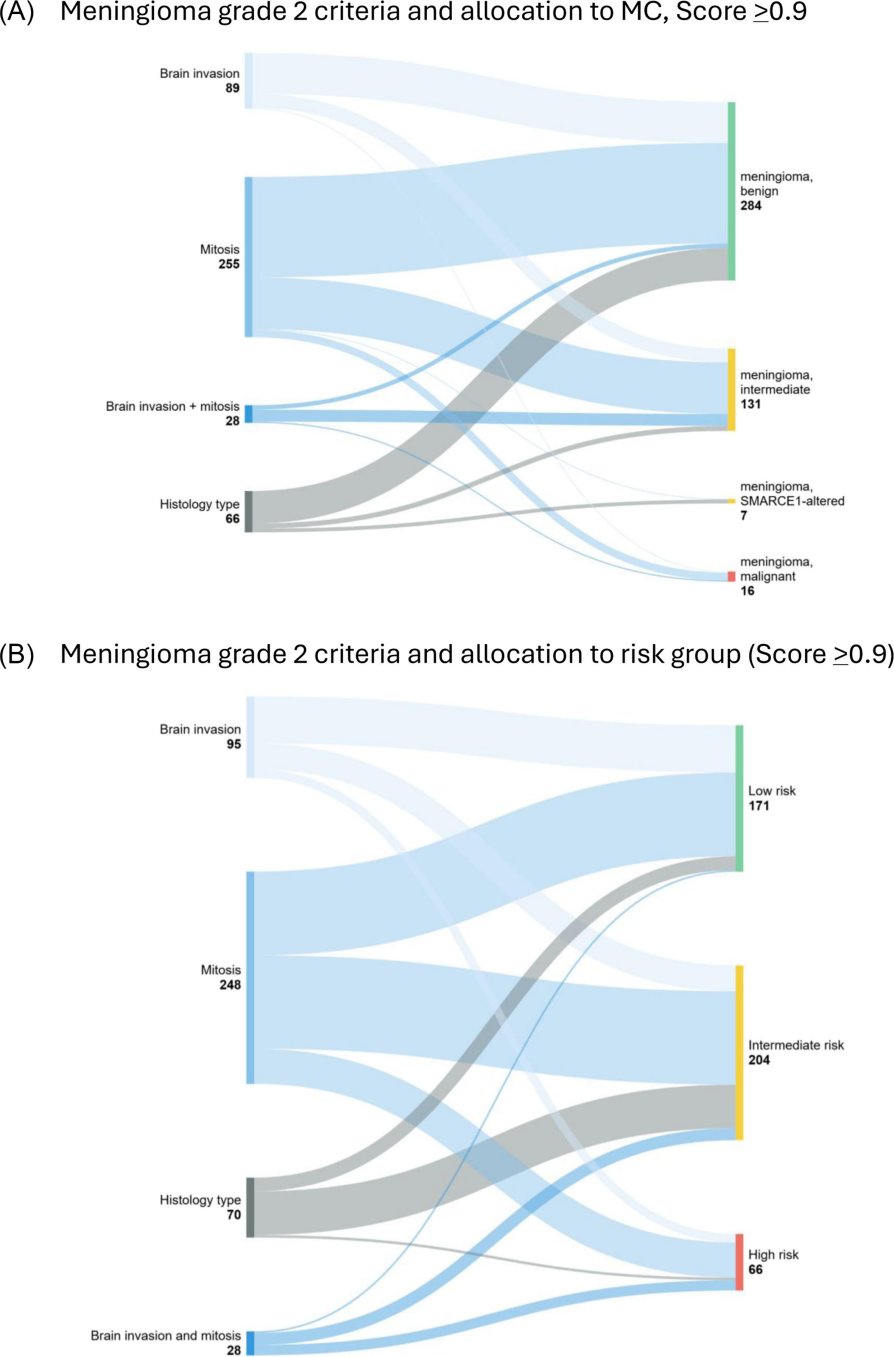
(A) Stratification of Grade 2 meningiomas by grading criteria (brain invasion, mitotic counts and histology type), and the proportional allocation into MCs benign, intermediate and malignant. Brain invasion and mitotic count show a similar distribution to MCs benign and intermediate, whereas tumours with both increased mitotic count and brain invasion predominantly allocate to the intermediate‐risk group. A majority of meningiomas with histological appearances suggesting Grade 2 were allocated to the benign MC. See also Table 1 for corresponding data. (B) Stratification of Grade 2 meningiomas by grading criteria (brain invasion, mitotic counts and histology type) and the allocation to the risk groups. The criteria brain invasion or mitotic activity distribute similarly into the three risk groups, with histology type predominantly into the intermediate‐risk group. See also Table 2 for corresponding data.

We established how these histological grading criteria were allocated to the MCs ‘benign’, ‘intermediate’ or ‘malignant’ or how these criteria resolved into the model scores of 0–9 (Figure 4A and Table 1). Four hundred forty‐one tumours were diagnosed as Grade 2, atypical meningiomas. Mitotic activity was the most frequent criterion (247/441, 58%), followed by brain invasion (95/441, 20%) and histology characteristics (55/441, 16%). Twenty‐eight atypical Grade 2 meningiomas showed brain invasion combined with increased mitotic activity (4–19/HPF) (28/441, 6%). However, the proportion of meningiomas resolving into the class ‘benign’ was similar for brain invasion (73%), increased mitoses (63%) and histology type (79%). Likewise, 25% of meningiomas with brain invasion and 32% of meningiomas with increased mitotic activity were allocated to the class ‘intermediate’. Instead, the MC ‘meningioma, malignant’ comprised two tumours with brain invasion and 12 tumours with increased mitotic count. Meningiomas with brain invasion and increased mitotic activity (4 and more mitoses per 10 HPF), mostly resolved into the MC ‘intermediate’ (*n* = 20, 68% of this group), with much less frequent allocation to the ‘benign’ class (*n* = 6, 21%) or ‘malignant’ (*n* = 2, 7%). Therefore, it can be concluded that the histological grading criteria (in particular, brain invasion and/or mitotic count) show no diagnostically relevant difference in terms of allocation to the MCs ‘benign’ or ‘intermediate’ (Figure 4A and Table 1).

**TABLE 1:** Atypical meningiomas, CNS WHO Grade 2, their grading criteria and allocation to methylation classes.

Grade 2 criteria	Methylation class				
	Meningioma, benign	Meningioma, intermediate	Meningioma, *SMARCE1*‐altered	Meningioma, malignant	Proportion of total
Brain invasion	69	24		2	**95**
Mitotic count	154	80	1	12	**247**
Histology type	55	9	6		**70**
Brain invasion + mitoses	7	20		2	**28**
Total	**285**	**133**	**7**	**16**	**441**
					
Brain invasion	0.73	0.25	0.00	0.02	**0.22**
Mitotic count	0.63	0.32	0.00	0.05	**0.56**
Histology type	0.79	0.13	0.09	0.00	**0.16**
Brain invasion + mitoses	0.21	0.71	0.00	0.07	**0.06**
Proportion of total	**0.65**	**0.30**	**0.02**	**0.04**	

### 3.5 Allocation of CNS WHO Grade 2 Meningioma Into Model Scores

**FIGURE 5:**
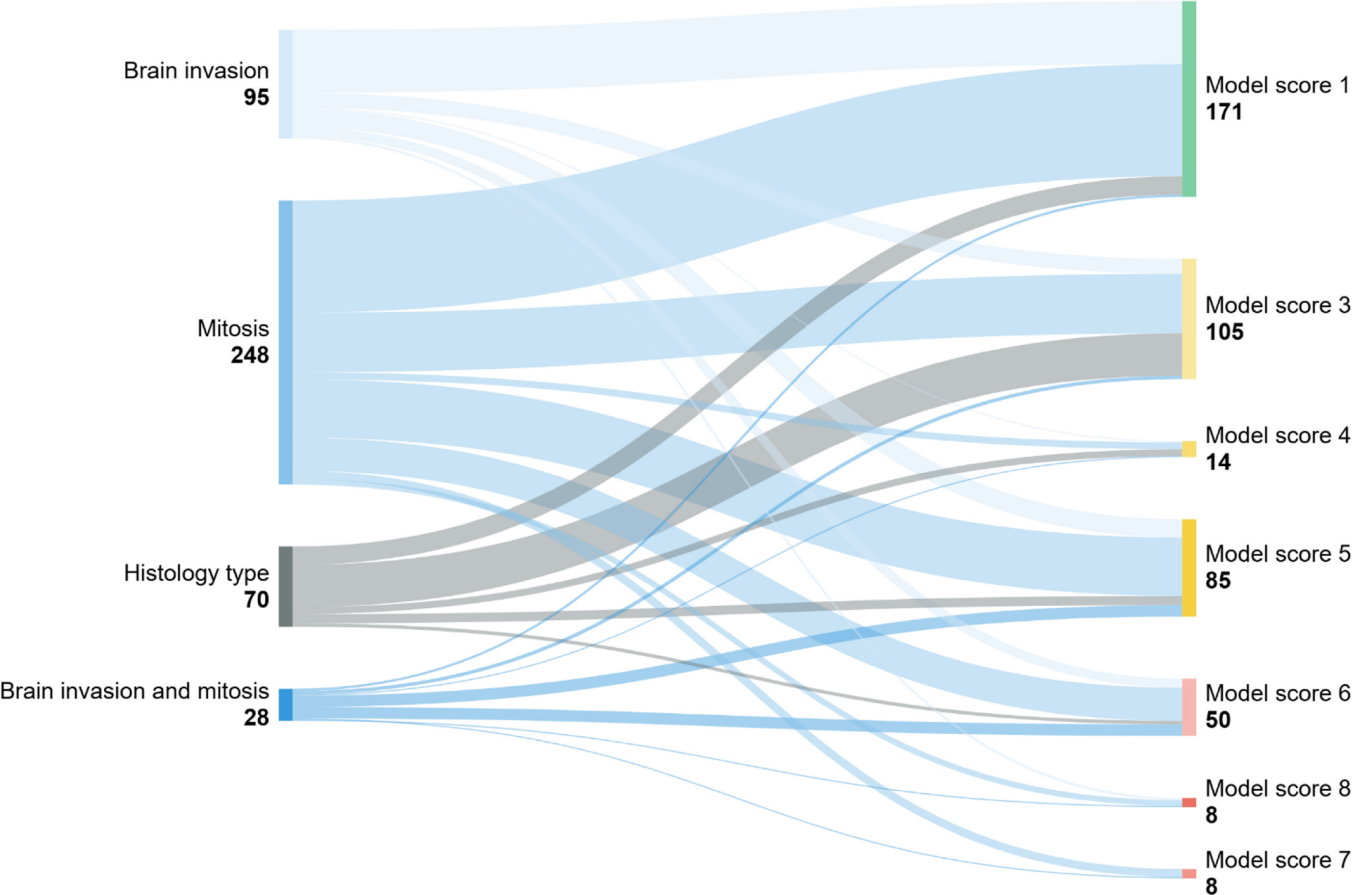
Stratification of Grade 2 meningiomas by grading criteria (brain invasion, mitotic counts and histology type) and the allocation to the individual model scores. See also Table S6 for corresponding data.

Next, we analysed how the histological Grade 2 criteria resolved into risk groups and the individual model scores (Figure 4B and Tables 2 and S6). Atypical meningiomas, Grade 2 due to brain invasion (*n* = 95/441, 22%) predominantly allocated to the low‐risk group with model scores of 0–2 (55/95, 58%), less frequently (30/95, 32%) to the intermediate‐risk group (scores of 3–5) and to a lesser extent (10/55, 11%) to the high‐risk group. In contrast, atypical meningiomas due to increased mitotic count (*n* = 247/441, 56%) were allocated similarly to the low‐risk group (98/247, 40%) or the intermediate‐risk group (109/247, 44%). 41/255 (17%) allocated to the high‐risk group. Most atypical meningiomas with brain invasion and increased mitotic activity (*n* = 28/438, 6%) were allocated to the intermediate‐risk group (14/28, 46%) and the high‐risk group (*n* = 12/28, 43%) but infrequently to the low‐risk group (2/28, 7%) (Figure 4B and Table 2). Figure 5 and Table S6 show the allocation of the histological grading criteria to the individual model scores. As expected from the calculation of the model scores, the minimum model score is ‘1’, due to the contribution of the WHO grade, and no score ‘2’ exists, as either the MC or the loss of chromosomes contributes at least 2 score points, i.e., result in a score of ‘3’. Furthermore, no score ‘9’ can exist in Grade 2 tumours. Grade 2 meningiomas predominantly spread across model scores of 1, 3 and 5 but were represented across all applicable scores.

**TABLE 2:** Atypical meningiomas, CNS WHO Grade 2, their grading criteria and allocation to risk groups (low, intermediate and high).

Grade 2 criteria	Risk group			
	Low risk	Intermediate risk	High risk	Total of criteria
Brain invasion	55	30	10	**95**
Mitotic count	98	109	41	**248**
Histology type	16	51	3	**70**
Brain invasion + mitoses	2	14	12	**28**
Total of risk groups	**171**	**204**	**66**	**441**
				Proportion of total
Brain invasion	0.58	0.32	0.11	**0.22**
Mitotic count	0.40	0.44	0.17	**0.56**
Histology type	0.23	0.73	0.04	**0.16**
Brain invasion + mitoses	0.07	0.50	0.43	**0.06**
Proportion of total (440)	**0.39**	**0.46**	**0.15**	

### 3.6 Analysis of the Relationship of Mitotic Counts With Model Scores and *CDKN2A/B* Status

The assessment of mitotic activity in meningioma is a central criterion in establishing the tumour grade. The mitotic counts qualifying for Grade 2 have a fairly wide range (4–19/10 HPF). Here, we analysed how mitotic count correlates with the model score or the *CDKN2A/B* status. We included meningiomas of all grades (Table S7) or specifically analysed atypical meningiomas whose grade was based on mitotic count only (Table S8). Mitotic counts from 711 Grade 1, 2 and 3 meningiomas that had undergone methylation profiling and yielded a calibrated score of 0.9 and higher were available from histological reports. Ungraded meningiomas were excluded from this analysis. Mitotic counts from Grade 1 meningiomas were reported either with a specific number of mitotic figures in 10 HPF or were expressed as ‘below 4/10 HPF’, as ‘inconspicuous’ or as ‘not significant’. Numerical mitotic counts were available from reports of meningiomas that were grade CNS WHO 2 due to mitotic activity. Reports from Grade 3 meningiomas indicated either the mitotic count or stated, ‘20 or more/10 HPF’. For analysis (Figures S3 and S4A,B), the following groups (strata for mitoses), with the respective frequencies, were established: 0–3 (*n* = 394), 4–7 (*n* = 234), 8–11 (*n* = 38), 12–15 (*n* = 14), 16–19 (*n* = 13) and 20–30 (*n* = 18) (Figure S3 and Table S7). As expected, mitotic activity correlates with the calculated risk score. Although the majority of meningiomas with 0–3 mitoses per 10 HPF (*n* = 394) were allocated to the low‐risk groups (*n* = 298, 76%), 88 (22%) were in the intermediate‐risk groups, and 8 (2%) were in the high‐risk groups. A mitotic count of 8–11/10 HPF (*n* = 38) is a fairly good predictor for an allocation into an intermediate‐ (53%) or high‐risk group (34%), and tumours with 12 and more mitotic figures per 10 HPF (*n* = 45) are never allocated to a low‐risk group, 13/45 (29%) to a intermediate‐risk group and a significant majority (32/45, 71%) to a high‐risk group. A separate analysis of the subset of Grade 2 meningiomas (based on mitotic count) and their allocation to model scores shows that scores of 1, 3 and 4 predominantly have low mitotic counts (Figure S3B and Table S8). Next, we analysed how the mitotic count correlates with the *CDKN2A/B* status (Figure S4C) [18]. The *CDKN2A/B* status was determined from the chromosome copy number read‐out of the methylation array and is a quantitative value. There is a modest correlation (Pearson coefficient −0.38) between mitotic count and *CDKN2A/B* status. Notably, *CDKN2A/B* deletions can be identified in tumours with as few as 6 or more mitotic figures per 10 HPF, but tumours with high mitotic counts may not necessarily have *CDKN2A/B* deletion.

### 3.7 Relationship Between Model Scores, Age Groups and Biological Sex

Meningiomas occur more frequently in females [3], with a reported female‐to‐male (F:M) ratio of 2.32:1, whereas high‐grade meningiomas preferentially affect males (F:M ratio 0.89:1) [1, 19, 20]. Consistent with published demographic data [1, 19, 20], our data show in Grade 1 meningiomas an F:M ratio of 2.4:1, in Grade 2 meningiomas an F:M ratio of 1. 6:1 and in Grade 3 meningiomas an F:M ratio of 0.7:1 (Figure S5A). The same trend can be seen in a more granular fashion by plotting the F:M ratio against the model score, where the F:M ratio is 3.6:1 at a model score of 0.5:1 at Score 8 (Figure S5B and Table S9). Figures S6 and S7 show stratification by age (bins of 10 years), with a low proportion of high‐risk meningiomas in younger females and an increase in the proportion of higher risk meningiomas in older females and the predominance of high‐risk meningiomas in males (Score 6: F:M 1:1 [*n* = 27:27], Score 7: F:M 0.4:1 [*n* = 6:9], Score 8: F:M 0.33:1 [*n* = 5:10] and only male patients with Score 9 [*n* = 3]).

### 3.8 Chromosome 22q Deletions in Meningiomas

Meningiomas commonly show a deletion of chromosome 22q, the frequency of which increases with the tumour grade. We have analysed the frequency in our cohort, based on meningiomas that underwent methylation profiling and classified with a calibrated score of 0.9 and higher in the classifier 12.6. In keeping with previous publications, any chromosomal loss exceeding 5% of the chromosomal arm was considered a ‘loss’. Nine hundred ninety meningiomas were included in this part of the analysis (Figure S8A and Table S2). In the entire cohort, 600 tumours had 22q loss, corresponding to 61%. Grade 1 meningiomas showed a loss in 52% (235/455), Grade 2 meningiomas showed a loss in 69% (303/442), and all Grade 3 meningiomas (*n* = 27) showed a loss. Ungraded meningiomas (due to either a small sample size or poor morphologic preservation) showed chromosome 22q loss (35/66, 53%), similar to the Grade 1 meningiomas. These data are consistent with previously published data and add useful additional information for future meta‐analyses. Correspondingly, the loss of chromosome 22q increases with higher model scores. In the group of meningiomas with a model score of 0 and 1, the proportion of tumours with 22q loss is lower, with 39% and 43%, respectively. Instead, within score Groups 5 and 6, there are only 4% of tumours with intact chromosome 22q (visually confirmed on the copy number plot), and no tumours in score Groups 7, 8 and 9 had intact chromosome 22q (Figure S8B,C).

### 3.9 Chromosome 1p and 22q Status in Meningiomas

**FIGURE 6:**
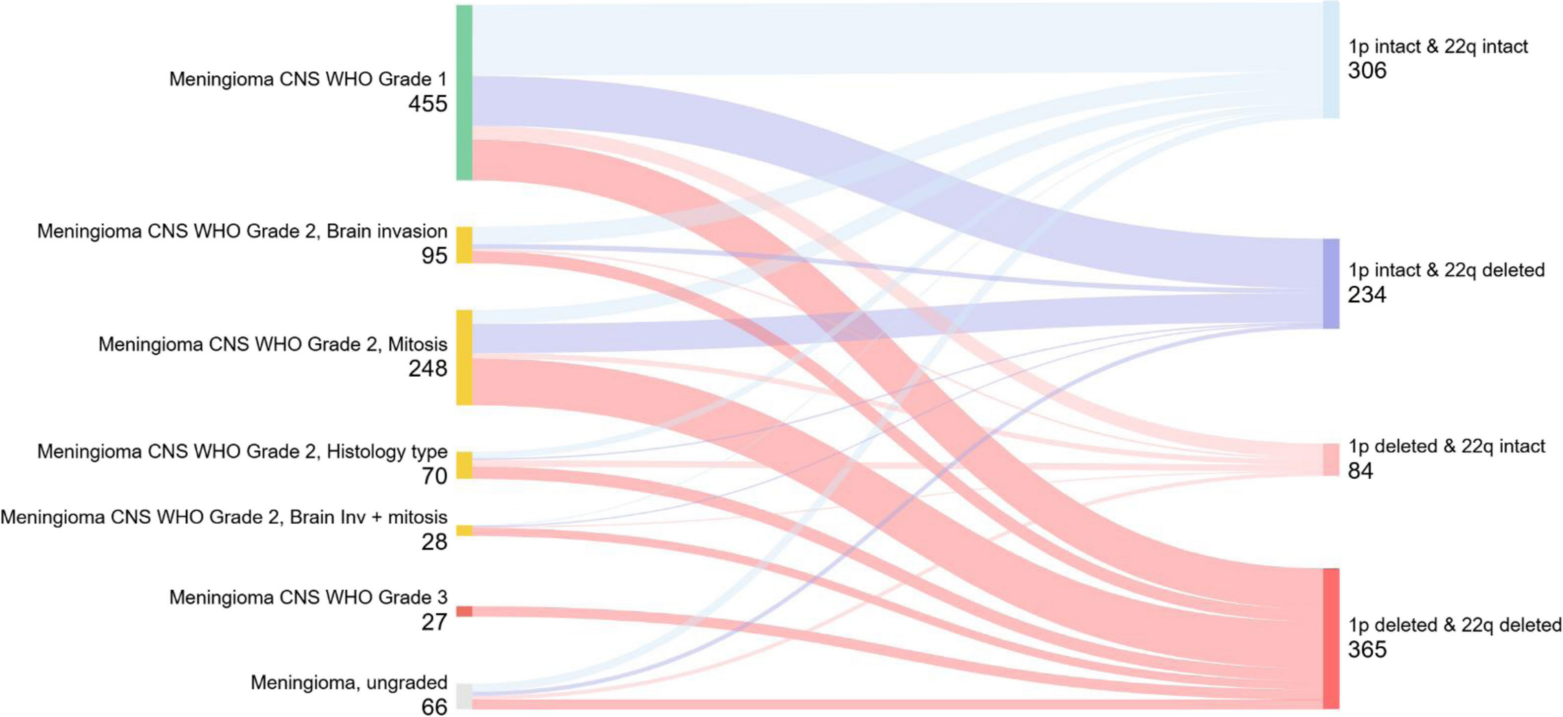
Contribution of Grade 1, 2 and 3 meningiomas to the group of 1p deleted or 1p intact meningiomas. See also Table S10 for corresponding data.

The recent publication of the cIMPACT‐NOW Update 8 [15] highlights the importance of analysis for chromosome 1p loss as a risk factor for progression. Specific analysis of 1p deletion (i.e., loss of more than 5% of chromosomal arm 1p) shows that Grade 1 and Grade 2 meningiomas contribute approximately equally to 1p‐intact meningiomas. Instead, a smaller fraction of Grade 1 meningiomas contributes to the group of 1p deleted tumours (Figure 6 and Table S10). To evaluate the frequencies of not only 1p deletion but also the concomitant 22q deletion, we have segregated our dataset into four groups: (1) 1p intact and 22q intact, (2) 1p intact and 22q deleted, (3) 1p deleted and 22q intact and (4) 1p deleted and 22q deleted. Our dataset shows the following key results: 142 of 455 Grade 1 meningiomas (31%) show a 1p deletion, and notably, within the group of the 142 1p‐deleted meningiomas, 36 (25%) had a concomitant intact chromosome 22q and are therefore more common than generally assumed [13]. Across all Grade 2 meningiomas, 56% (246/441) had a 1p deletion (regardless of 22q status), and within this group, the absolute contribution of 1p deletion within each subgroup was 37/95 (39%) within the brain invasive, 136/247 (55%) in the mitotically active, 49/70 (70%) in the histology‐type group and 24/28 (86%) in the brain invasive + mitosis group. All meningiomas with the histological criteria of Grade 3 were 1p and 22q deleted. Nineteen percent of 1p deleted meningiomas had an intact 22q chromosome.

## 4 Discussion

Our study analysed a large cohort of meningiomas in adults, diagnosed between January 2017 and December 2023 in our centre or referred from other centres. We analysed meningiomas that underwent methylation array analysis to establish a methylation profile [9] or, after the publication of follow‐up studies [12, 16], to calculate the model score. During this period, we observed a continuous increase in requests for methylation profiling, due to the availability of increasingly sophisticated algorithms to predict the risk of early recurrence, and the recognition of the clinical and diagnostic importance of risk stratification [4, 9, 11, 12, 14, 15, 16, 21, 22]. To avoid bias, only the cohort of internally diagnosed tumours has been considered, as referred tumours would have presented a significant preselection bias through the referral practice. At the point of decision‐making, whether or not to profile a tumour, only the WHO grade determined by histological features, the patient's biological sex and age and tumour location were available. Approximately 90% of Grade 1 meningiomas were allocated to the MC ‘benign’ and the remaining 10% to the MC ‘intermediate’ or, in a single case, to the class of *SMARCE1*‐altered. Likewise, nearly 90% of Grade 1 meningiomas also allocated to the low‐risk model scores of 0, 1 and 2, and all other Grade 1 meningiomas into the intermediate‐risk Groups 3, 4 and 5, but none of these to the higher risk Groups 6–9. Instead, Grade 2 meningiomas showed an allocation to the MCs ‘benign’ (approximately two‐thirds), ‘intermediate’ (just over one‐third), ‘malignant’ (3%) and *SMARCE1*‐altered (1.6%) (Figure 3 and Table S3). In keeping, there was a similarly broad allocation across risk scores, spanning from Score 1 up to Score 8 (Figure 4 and Tables S3 and S5). Meningiomas were graded as anaplastic (Grade 3), classified as ‘benign’, ‘intermediate’ and ‘malignant’ at approximately similar proportions, with model scores between 4 and 9.

Our analysis of how histological criteria leading to Grade 2 allocate to MCs or model scores shows that for each criterion, the proportion of tumours allocating to the classes ‘benign’, ‘intermediate’ or ‘malignant’ is similar to the proportions across all Grade 2 meningiomas (Figure 4A and data in Table S6). The only exception is Grade 2 meningiomas with increased mitotic count combined with brain invasion, which is allocated predominantly to the MC ‘intermediate’. In keeping, Grade 2 meningiomas are similarly allocated to the risk groups ‘low’, ‘intermediate’ and ‘high’ (Figure 4B) and to the individual model scores Figure 5. In conclusion, Grade 2 meningiomas benefit most from additional analysis by methylation profiling, as the outcomes of the risk stratification can vary considerably. This addresses some of the aspects raised in the cIMPACT‐NOW Update 8 [15]. Based on our data, a practical approach, given economic constraints, would be to prioritise molecular testing of CNS Grade 2 and 3 meningiomas.

With the grade as a key criterion to prioritise meningiomas for methylation profiling, mitotic count is essential and is mandated in pathology reports. Tumours with over 8 mitotic figures in 10 HPF are unlikely to be in a low‐risk stratum, and tumours with over 12 mitoses per 10 HPF were not allocated to a low‐risk group at all (Figure S3). Therefore, if economic constraints mandate further prioritisation, tumours with mitotic counts of over 12/10 HPF may not require risk stratification as they will always be allocated to intermediate‐ and high‐risk groups. However, in our cohort of 711 meningiomas, only 45 had mitotic counts over 12/10 HPF, and therefore, the exclusion from additional molecular testing is only of limited economic benefit. Furthermore, tumours with high mitotic counts frequently, but not predictably, show *CDKN2A/B* deletions (Figure S4), which is relevant for accurate grading. It remains, however, unresolved how to predict the 10% Grade 1 meningiomas that are allocated to the MC intermediate or risk scores of 3, 4 or 5. Perhaps in the future, with advanced image analysis algorithms, it may be possible to predict higher risks, including chromosome 1 loss, from whole slide digital images.

Ungraded meningiomas, i.e., tumours that are small, or with significant morphological alteration (thermocoagulation artefacts or crush artefacts), are very useful candidates for methylation profiling. Given the uncertainty in grade due to the limited morphological assessment, the added value of copy number profiling and determination of MC is relevant, as it can reassure clinical teams and ultimately the patient of an expected risk of recurrence, including reassurance of a benign nature in a majority (49/66, 74%) of cases in our cohort).

Our study further confirms established epidemiological data, such as an increased proportion of male patients in higher grade meningiomas (Figure S5A), and in keeping, the same trend of shifting towards a higher proportion of affected males from model scores of 0–9 (Figure S5B). Occurrence of meningiomas in young patients, mostly females, often prompts pathologists or clinicians to request methylation array–based risk stratification, but our data suggest a lower incidence of intermediate‐ or high‐risk scores in younger female patients and a higher incidence of high‐risk scores in females between 50 and 80 and in particular in males between 40 and 80 (Figures S6 and S7).

A surprising finding was the relatively small number of high‐risk meningiomas in young women, with an increase at higher ages. Although we would not necessarily advocate omitting testing of meningiomas in young females, our data do not suggest that high‐risk meningiomas are more likely in this patient group.

An important prognostic factor is the loss of chromosomal arm 1p in meningiomas, as ‘oncogenic phylogeny models propose 1p loss as the first CNV after monosomy 22q in NF2‐mutant, high‐grade meningiomas, with rare cases showing 1p loss in the absence of 22q loss (…)’ [15]. To evaluate the frequencies of not only 1p deletion but also concomitant 22q deletion, we segregated our dataset into four groups: 1p intact and 22q intact, 1p intact and 22q deleted, 1p deleted and 22q intact and 1p deleted and 22q deleted (Figure 6 and Table S10). Our dataset shows the following key results: (i) One‐third of Grade 1 meningiomas have a deleted chromosome 1p, (ii) approximately half of Grade 2 meningiomas have a deleted chromosome 1p, and (iii) nearly one‐fifth of all 1p deleted meningiomas had an intact chromosome 22q, which is noteworthy considering the established phylogenetic sequence of chromosomal aberrations in meningiomas [13]. These results, however, also make a stratification and recommendation as to which meningioma grades to prioritise for testing more challenging. Therefore, the identification of an affordable, robust and easy‐to‐test biomarker for 1p deletion, possibly by artificial intelligence–powered histological analysis, would be ideal to recognise meningiomas that should undergo methylation profiling. Analysis of brain invasive, otherwise benign (‘BIOB’) meningiomas showed that 42% were allocated to the intermediate model scores and 39% had a 1p deletion, suggestive of a higher risk of recurrence.

Our study has several limitations: (i) It comprises diagnostic cases from the last 6 years, and therefore, clinical outcome data are not available to validate the decision‐making process; (ii) there is incomplete availability of data on tumour location, i.e., our study cannot corroborate previous studies in which different methylation profiles are associated with tumour locations [9, 21]; (iii) there could be selection bias for Grade 1 meningiomas that underwent profiling; (iv) multiple approaches have been established to perform risk of early recurrence stratification on meningiomas, and only one such approach (methylation profiling and chromosome copy number calculation, with calculation of a model score) has been applied in this study. A further limitation is (v) the unavailability of *TERT* promoter mutation data [23, 24], as this is not routinely tested in Grade 1 or 2 meningiomas. The rationale for not testing *TERT* promoter mutations in meningiomas is the absence of *TERT* promoter mutations in low‐risk meningiomas [12, 25], and the integrated score based on the 2021 WHO classification is most accurate in risk prediction. Likewise, for similar reasons, the status of trimethylated histone H3 (H3 K27me3) was only rarely assessed. A large study identified H3 K27me3 loss in just under 5% of meningiomas, mostly in recurrent and in higher grade tumours, but even in anaplastic meningiomas, it reached only 20% [26, 27]. Although it is undisputed that H3 K27me3 loss is enriched in Grade 2 and 3 meningiomas, the sensitivity of this test is low, and therefore, in our practice, we rely on methylation profiling and do not perform additional H3 K27me3 staining. Retrospective testing would be possible but would be outside the scope of this study, which primarily aims at establishing a testing algorithm for meningiomas. H3 K27me3 staining may be of limited utility, as a recent study suggests no added value of *TERT* promoter sequencing beyond methylation analysis [12]; (vi) the classification result used in this study is limited to the MCs ‘benign’, ‘intermediate’ and ‘malignant’ but not the subclasses (ben‐1,2, 3; intermediate A, B); however, we reasoned that further granularity is unlikely to change the decision‐making process and the overall conclusion of the study; (vii) mitotic counts were expressed as mitotic figures per 10 HPF and were retrieved from histology reports from 2017 to 2023, and therefore, metric values cannot be provided but would need to be inferred. Reporting mitotic counts can vary between pathologists and thus may represent a bias. (viii) There is a general limitation that detection of brain invasion depends on the comprehensiveness of the analysed material, i.e., it may not always be represented in diagnostic samples, and this could present a bias in our study.

In conclusion, our study reinforces epidemiological findings and the prevalence of histological grades. It provides important data to guide the decision‐making process and supports the evidence to prioritise testing of Grade 2 and 3 meningiomas. Our study also underpins the importance of molecular analysis of brain invasive, otherwise benign meningiomas, which frequently carry chromosomal and epigenetic features suggestive of a higher risk of recurrence.

## Author Contributions

F.R. and S.B. conceived and designed the work. F.R., R.R., Z.J., A.M., L.A., M.D. and S.B. acquired, analysed and interpreted data. S.B., Z.J., A.M. and F.S. drafted the manuscript. S.B. and R.R. revised the manuscript. All authors read and approved the final manuscript.

## Ethics Statement

Ethical approval was obtained from BRAIN UK, reference number: 19/002—molecular analyses of adult brain tumours by conventional molecular tests and DNA methylation profiling. Data were obtained from University College London NHS Foundation Trust as part of the UK Brain Archive Information Network (BRAIN UK), which is funded by the Medical Research Council and Brain Tumour Research. All authors have seen the manuscript and have approved the publication. Anonymised datasets can be made available on request.

## Conflicts of Interest

Felix Sahm is a co‐founder and shareholder of Heidelberg Epignostix GMBH, which licences the use of the methylation classifier. The other authors declare no conflicts of interest. Felix Sahm, Zane Jaunmuktane and Sebastian Brandner are members of the editorial board at Neuropathology and Applied Neurobiology.

## Peer Review

The Editors of Neuropathology and Applied Neurobiology are committed to peer‐review integrity and upholding the highest standards of review. As such, this article was peer‐reviewed by independent, anonymous expert referees, and the authors had no role in either the editorial decision or the handling of the paper.

## Supporting information


**Figure S1** Proportion of meningiomas on which methylation array profiling and risk prediction were performed, direct referrals. Due to the nature of the request, nearly all referred tumours underwent methylation profiling. The corresponding data are in Table 2.
**Figure S2** Meningiomas grade and allocation to MCs (all profiled tumours with a calibrated score of 0.3 up to 0.9. The allocation of the WHO grades to the MCs benign, intermediate and malignant is similar to those with a calibrated score of 0.9 and above. The corresponding data are in Table 4.
**Figure S3** Granular analysis and allocation of the mitotic counts to the model scores of 0–9. Upper graph: All mitotic counts, i.e., of meningiomas Grades 1, 2 and 3. Lower graph: Allocation of Grade 2 meningiomas only, showing an allocation of higher mitotic counts towards higher model scores. The corresponding data are in Tables 9 (upper graph) and 10 (lower graph).
**Figure S4** Model score and corresponding mitotic counts of all meningiomas. The upper graph (A) shows the absolute frequency and the centre graph (B) the relative frequency. See also Table S7 for the corresponding data. (C) Mitotic count and CDKN2A/B status. X axis, mitotic count (expressed in mitoses per 10 HPF), Y axis CDKN2A/B value determined from the copy number plot. There is a weak correlation between mitotic count and CDKN1A/B status, with a Pearson coefficient of 0.38.
**Figure S5** Upper graph: Distribution of patients’ sex and grade of meningiomas. There is a preponderance of female sex (2.4:1) in Grade 1 meningiomas, a reduction of female representation in Grade 2 meningiomas (1.6:1) and a male predominance in Grade 3 meningiomas (0.7:1). Lower graph: In keeping with the decreasing female dominance with increasing grade, the same trend can be observed when plotting the sex distribution against the model score, showing an even stronger predominance of female sex in Score 0 tumours (3.6:1) and a stronger prevalence of male sex in higher scores, i.e., 0.7:1 in Score 7 and 0.5:1in Score 8 tumours. See Table S1 for the corresponding data.
**Figure S6** Distribution of model scores across patient age ranges (absolute numbers). Upper graph, male patients; lower graph, female patients. In keeping with the more frequent occurrence of high‐grade meningioma in males, this graph shows that the high‐risk groups are more represented in male patients. It also shows that meningiomas operated in young females do not have a higher proportion of intermediate‐ and high‐risk scores than in the middle‐aged and elderly population.
**Figure S7** Distribution of model scores across patient age ranges (as a fraction of 1 for each stratum). Upper graph, male patients; lower graph, female patients. In keeping with the more frequent occurrence of high‐grade meningioma in males, this graph shows that the high‐risk groups are more represented in male patients. It also shows that meningiomas operated in young females do not have a higher proportion of intermediate‐ and high‐risk scores than in the middle‐aged and elderly population.
**Figure S8** (A) Loss of chromosome 22q is seen in approximately 50% of Grade 1 tumours, and this proportion increases in Grade 2 tumours, whereas all Grade 3 tumours have a loss of chromosome 22q. The corresponding data are in Table S2. (B, C) Model score and corresponding mitotic counts of all meningiomas. The upper graph shows the absolute frequency and the lower graph the relative frequency. See also Table S7 for the corresponding data.

**Table S1** Meningiomas diagnosed internally, with or without array testing.
**Table S2** Meningiomas referred for a second opinion, mostly for methylation array profiling.
**Table S3** Meningiomas with methylation profile, with a calibrated score of 0.9–1.0. Only these meningiomas were included in the subsequent analysis.
**Table S4** Meningiomas with methylation profile, with a calibrated score of 0.3 < 0.9. These meningiomas were excluded from the analysis.
**Table S5** Diagnosed grade and their allocation to the model scores of 0–9. Only samples with calibrated scores of 0.9 and higher were included in the analysis (*n* = 924), and meningiomas with no initial WHO grade (*n* = 66) were excluded.
**Table S6** Atypical meningiomas, CNS WHO Grade 2, their grading criterium (left column) and allocation to model score (0–9, top row).
**Table S7** Mitotic counts, grouped into strata (0–3, 4–7, 8–11, 12–15, 16–19 and 20–30) and allocation to the model scores, including CNS WHO Grade 1, 2 and 3 meningiomas.
**Table S8** CNS WHO Grade 2 meningiomas which were graded based on mitotic count only, i.e., excluding histology characteristics or brain invasion. Mitotic counts are grouped into strata (4–7, 8–11, 12–15, 16–20) and allocated to the model scores of 1–8.
**Table S9** Male‐to‐female ratios increase with higher model scores.
**Table S10** Chromosome 1p status in meningiomas of CNS WHO Grades 1, 2 and 3 with existing chromosome 22q deletion (> 5% of the chromosome) or intact chr 22q. For CNS WHO Grade 2 meningiomas, the data are separated by grading criteria (BI = brain invasion, Mit = mitotic counts, Histo = histological type, BI + Mit = brain invasion and increased mitotic count). See Figure 9 for the corresponding illustration.
**Table S11**: Chromosome 22q status and initial WHO grade in 990 meningiomas with a calibrated score of 0.9 and higher.
**Table S12**: Chromosome 22q status and model score, in 990 meningiomas with a calibrated score of 0.9 and higher.

## Data Availability

The data that support the findings of this study are available on request from the corresponding author. The data are not publicly available due to privacy or ethical restrictions.
