# Supplementary material for: Testing Meningiomas With Methylation Arrays: Insights and Recommendations From a Large Single‐Centre Study
Source: Neuropathol Appl Neurobiol. 2025 May 13;51(3):e70018. doi: 10.1111/nan.70018 (PMC12070139; doi:10.1111/nan.70018)
Supplement: Supplementary file 1 — Figure S1 Proportion of meningiomas on which methylation array profiling and risk prediction were performed, direct referrals. Due to the nature of the request, nearly all referred tumours underwent methylation profiling. The corresponding data are in Table 2. Figure S2 Meningiomas grade and allocation to MCs (all profiled tumours with a calibrated score of 0.3 up to 0.9. The allocation of the WHO grades to the MCs benign, intermediate and malignant is similar to those with a calibrated score of 0.9 and above. The corresponding data are in Table 4. Figure S3 Granular analysis and allocation of the mitotic counts to the model scores of 0–9. Upper graph: All mitotic counts, i.e., of meningiomas Grades 1, 2 and 3. Lower graph: Allocation of Grade 2 meningiomas only, showing an allocation of higher mitotic counts towards higher model scores. The corresponding data are in Tables 9 (upper graph) and 10 (lower graph). Figure S4 Model score and corresponding mitotic counts of all meningiomas. The upper graph (A) shows the absolute frequency and the centre graph (B) the relative frequency. See also Table S7 for the corresponding data. (C) Mitotic count and CDKN2A/B status. X axis, mitotic count (expressed in mitoses per 10 HPF), Y axis CDKN2A/B value determined from the copy number plot. There is a weak correlation between mitotic count and CDKN1A/B status, with a Pearson coefficient of 0.38. Figure S5 Upper graph: Distribution of patients’ sex and grade of meningiomas. There is a preponderance of female sex (2.4:1) in Grade 1 meningiomas, a reduction of female representation in Grade 2 meningiomas (1.6:1) and a male predominance in Grade 3 meningiomas (0.7:1). Lower graph: In keeping with the decreasing female dominance with increasing grade, the same trend can be observed when plotting the sex distribution against the model score, showing an even stronger predominance of female sex in Score 0 tumours (3.6:1) and a stronger prevalence of male sex in higher scores, i.e., 0.7 [file NAN-51-e70018-s002.pptx]

## Slide 1
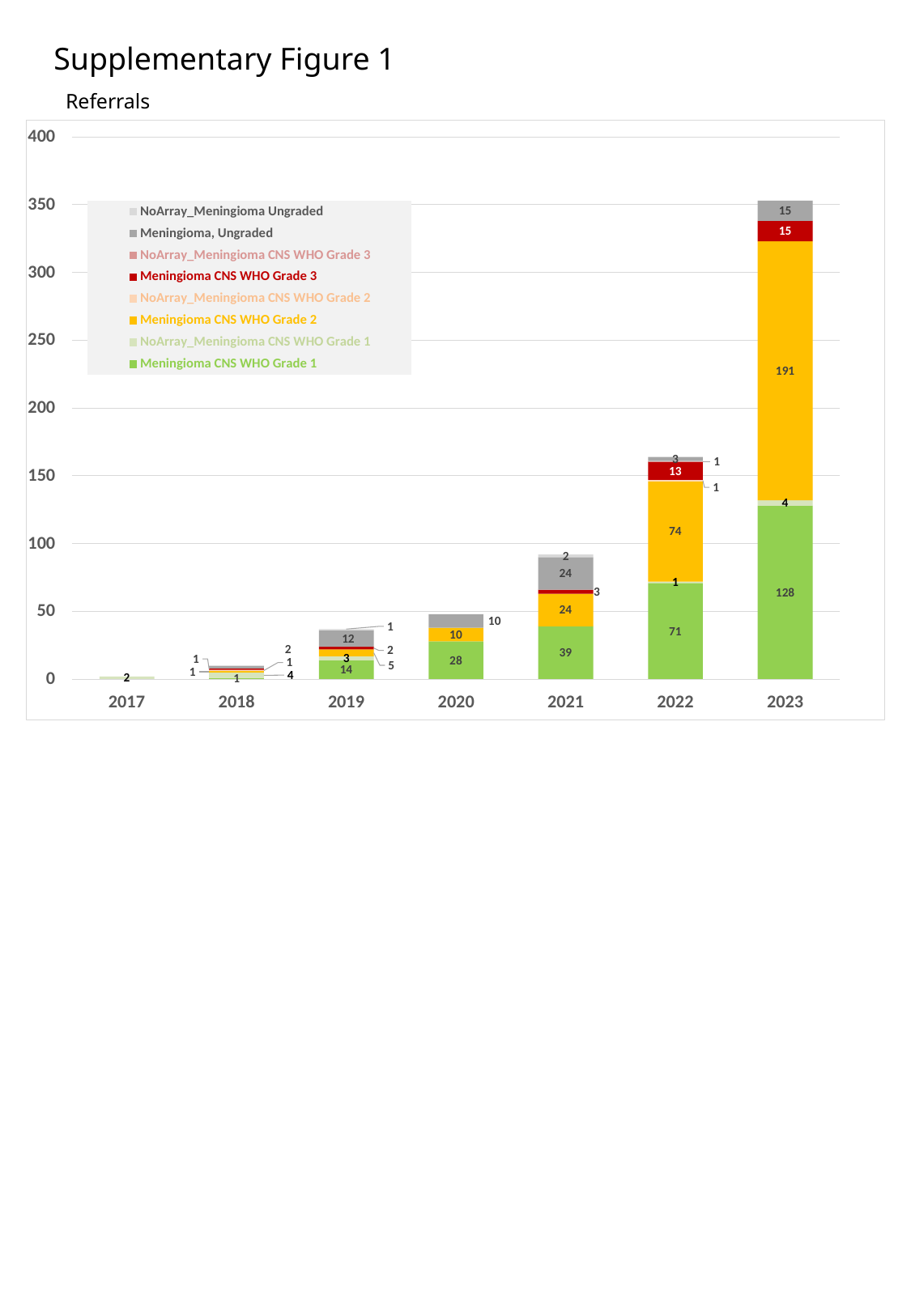

Supplementary Figure 1
Referrals
### Chart
| Category | Meningioma CNS WHO Grade 1 | NoArray_Meningioma CNS WHO Grade 1 | Meningioma CNS WHO Grade 2 | NoArray_Meningioma CNS WHO Grade 2 | Meningioma CNS WHO Grade 3 | NoArray_Meningioma CNS WHO Grade 3 | Meningioma, Ungraded | NoArray_Meningioma Ungraded |
|---|---|---|---|---|---|---|---|---|
| 2017 | None | 2.0 | None | None | None | None | None | None |
| 2018 | 1.0 | 4.0 | 1.0 | 1.0 | 1.0 | None | 2.0 | None |
| 2019 | 14.0 | 3.0 | 5.0 | None | 2.0 | None | 12.0 | 1.0 |
| 2020 | 28.0 | None | 10.0 | None | None | None | 10.0 | None |
| 2021 | 39.0 | None | 24.0 | None | 3.0 | None | 24.0 | 2.0 |
| 2022 | 71.0 | 1.0 | 74.0 | 1.0 | 13.0 | 1.0 | 3.0 | None |
| 2023 | 128.0 | 4.0 | 191.0 | None | 15.0 | None | 15.0 | None |

## Slide 2
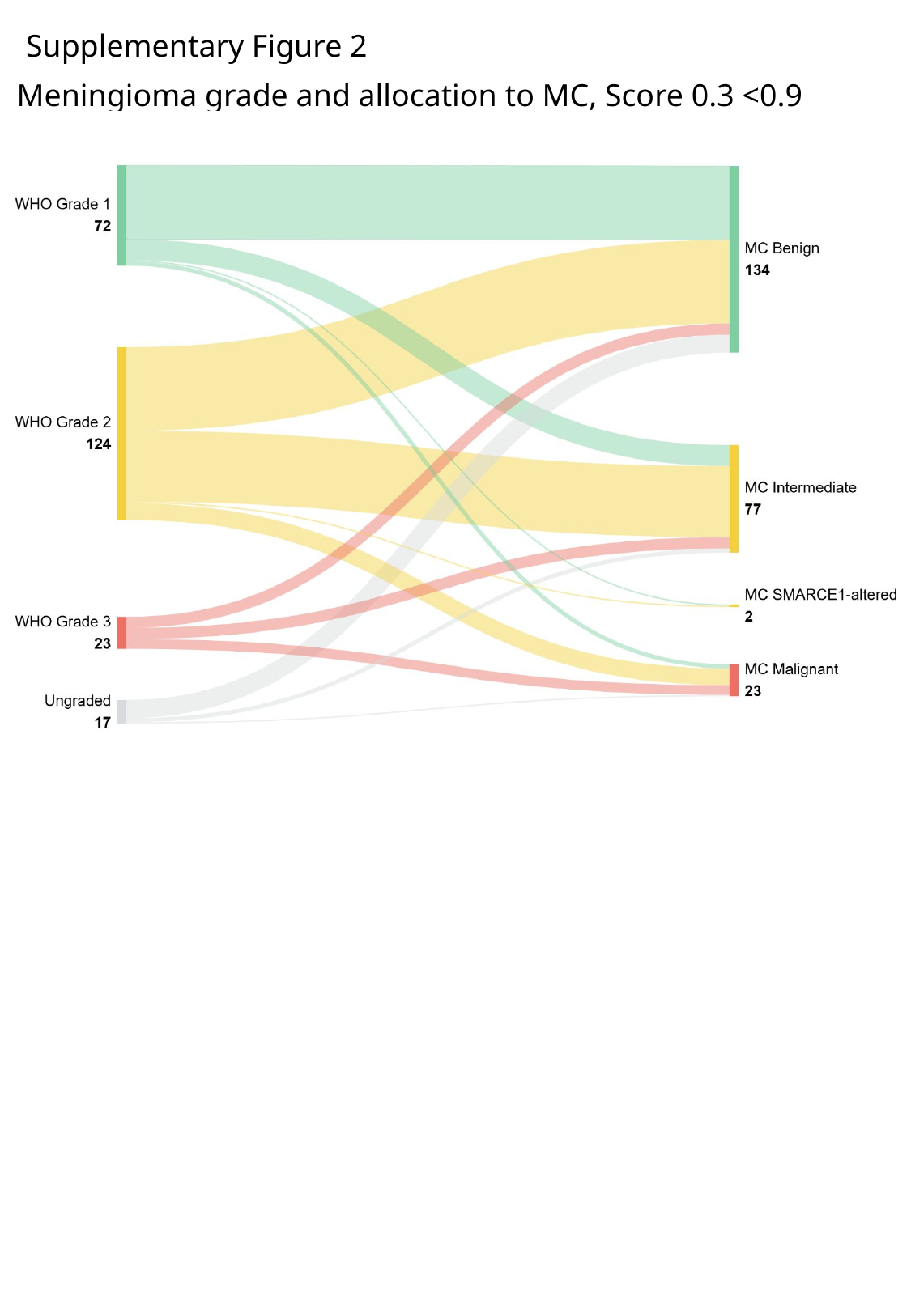

Supplementary Figure 2
Meningioma grade and allocation to MC, Score 0.3 <0.9

## Slide 3
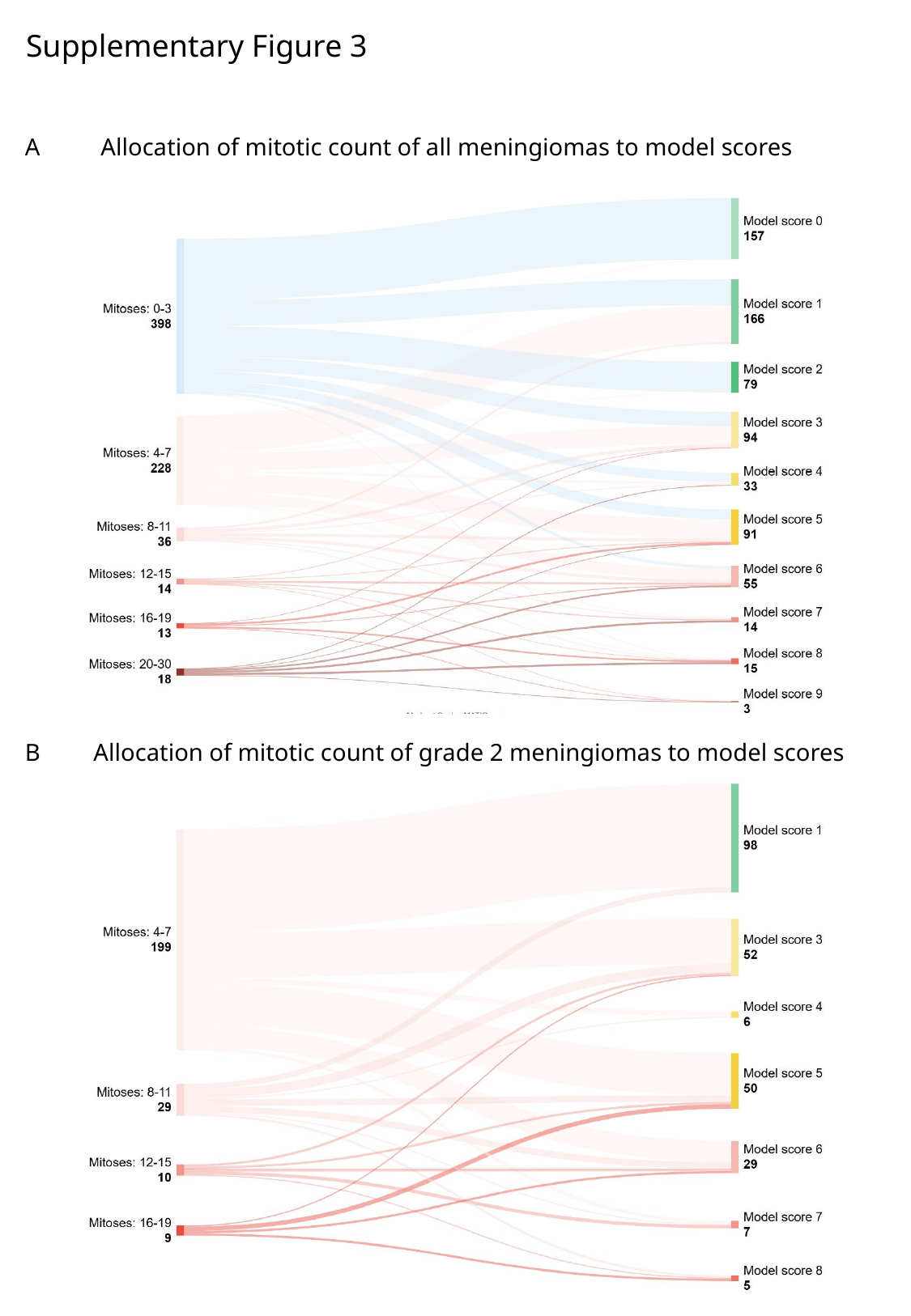

Supplementary Figure 3
A
Allocation of mitotic count of all meningiomas to model scores
B
Allocation of mitotic count of grade 2 meningiomas to model scores

## Slide 4
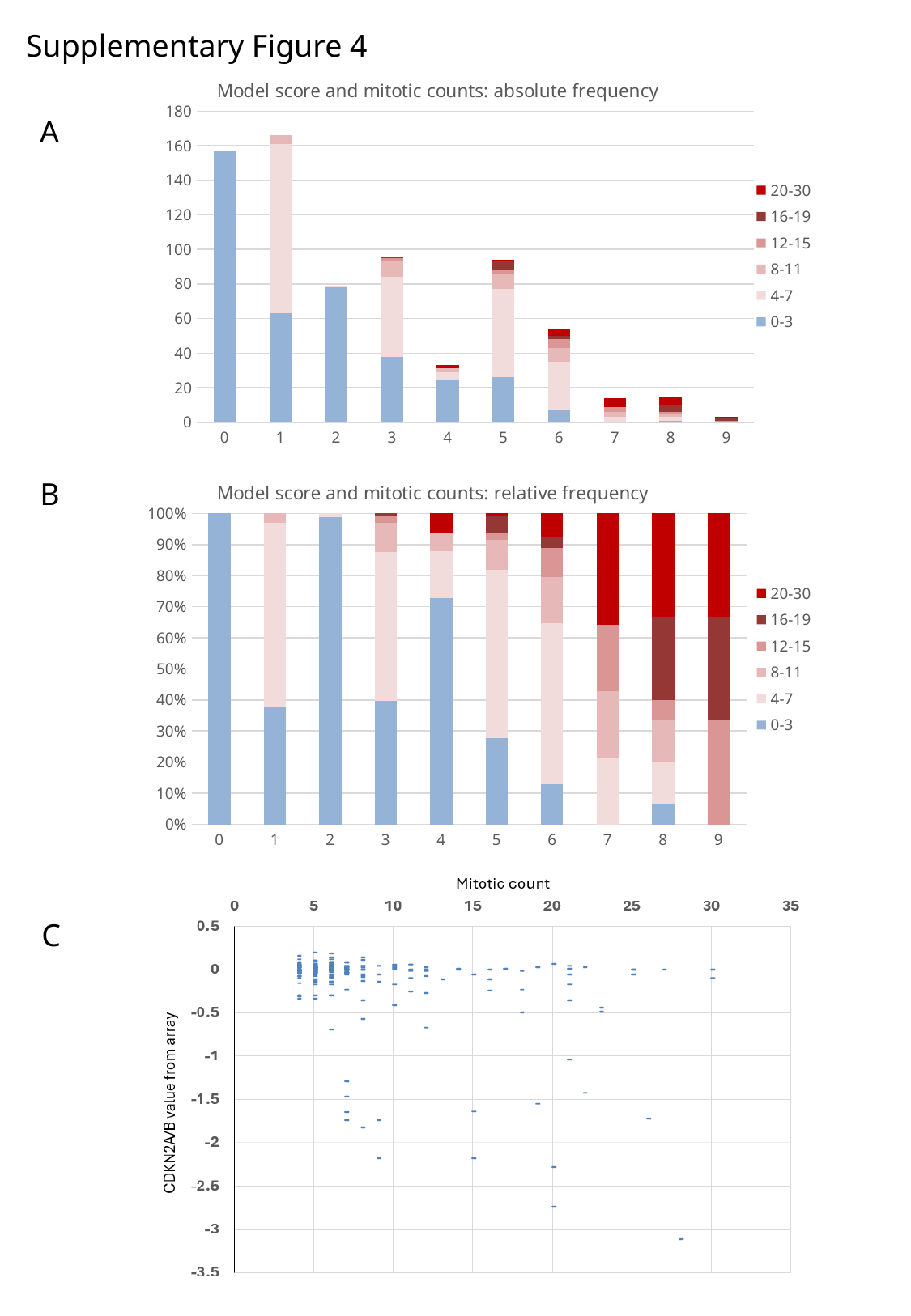

Supplementary Figure 4
### Chart: Model score and mitotic counts: absolute frequency
| Category | 0-3 | 4-7 | 8-11 | 12-15 | 16-19 | 20-30 |
|---|---|---|---|---|---|---|
| 0 | 157.0 | None | None | None | None | None |
| 1 | 63.0 | 98.0 | 5.0 | None | None | None |
| 2 | 78.0 | 1.0 | None | None | None | None |
| 3 | 38.0 | 46.0 | 9.0 | 2.0 | 1.0 | None |
| 4 | 24.0 | 5.0 | 2.0 | None | None | 2.0 |
| 5 | 26.0 | 51.0 | 9.0 | 2.0 | 5.0 | 1.0 |
| 6 | 7.0 | 28.0 | 8.0 | 5.0 | 2.0 | 4.0 |
| 7 | None | 3.0 | 3.0 | 3.0 | None | 5.0 |
| 8 | 1.0 | 2.0 | 2.0 | 1.0 | 4.0 | 5.0 |
| 9 | None | None | None | 1.0 | 1.0 | 1.0 |A
### Chart: Model score and mitotic counts: relative frequency
| Category | 0-3 | 4-7 | 8-11 | 12-15 | 16-19 | 20-30 |
|---|---|---|---|---|---|---|
| 0 | 157.0 | None | None | None | None | None |
| 1 | 63.0 | 98.0 | 5.0 | None | None | None |
| 2 | 78.0 | 1.0 | None | None | None | None |
| 3 | 38.0 | 46.0 | 9.0 | 2.0 | 1.0 | None |
| 4 | 24.0 | 5.0 | 2.0 | None | None | 2.0 |
| 5 | 26.0 | 51.0 | 9.0 | 2.0 | 5.0 | 1.0 |
| 6 | 7.0 | 28.0 | 8.0 | 5.0 | 2.0 | 4.0 |
| 7 | None | 3.0 | 3.0 | 3.0 | None | 5.0 |
| 8 | 1.0 | 2.0 | 2.0 | 1.0 | 4.0 | 5.0 |
| 9 | None | None | None | 1.0 | 1.0 | 1.0 |B
C

## Slide 5
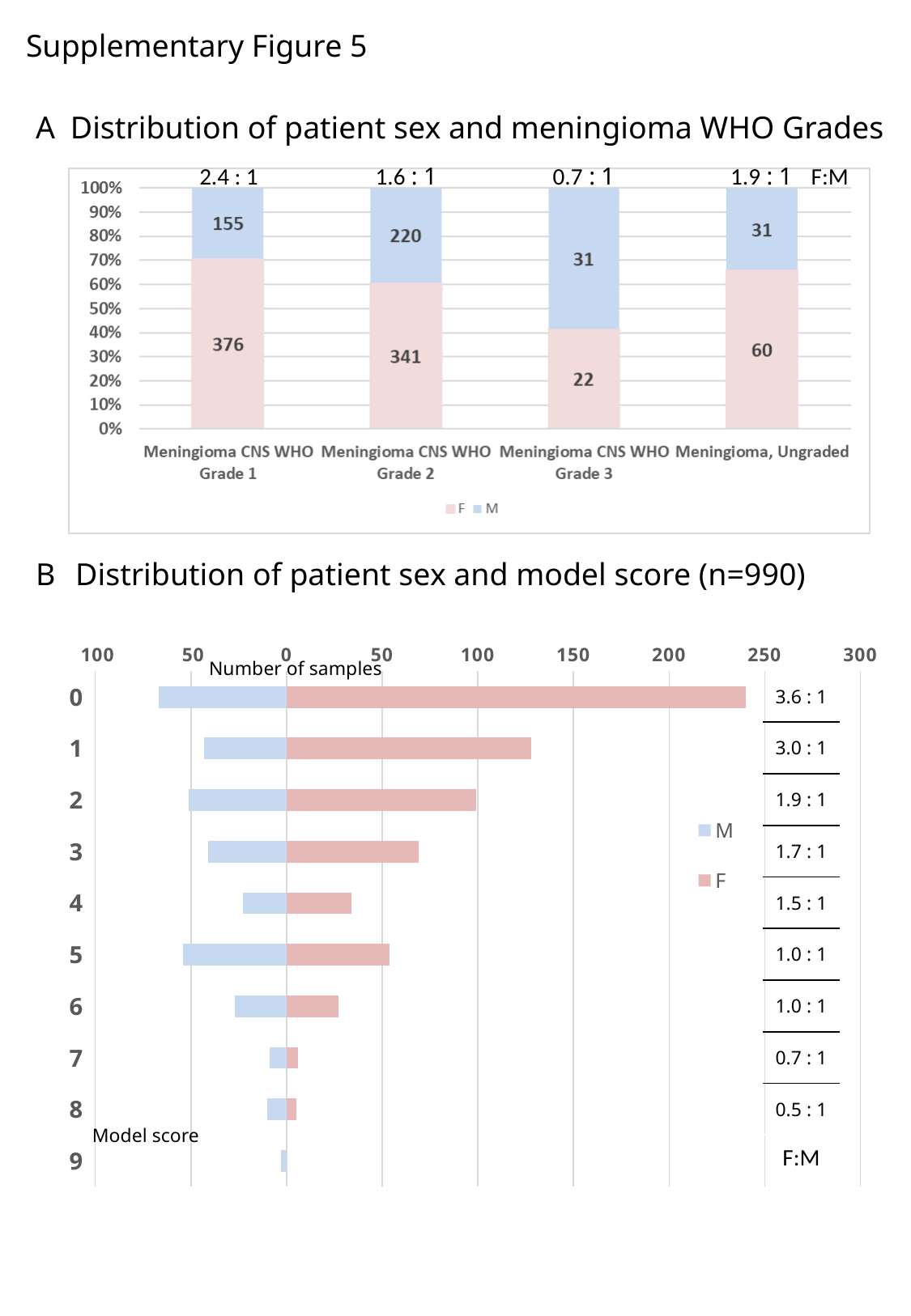

Supplementary Figure 5
A
Distribution of patient sex and meningioma WHO Grades
2.4 : 1
1.6 : 1
0.7 : 1
1.9 : 1
F:M
B
Distribution of patient sex and model score (n=990)
### Chart
| Category | F | M |
|---|---|---|
| 0 | 240.0 | -67.0 |
| 1 | 128.0 | -43.0 |
| 2 | 99.0 | -51.0 |
| 3 | 69.0 | -41.0 |
| 4 | 34.0 | -23.0 |
| 5 | 54.0 | -54.0 |
| 6 | 27.0 | -27.0 |
| 7 | 6.0 | -9.0 |
| 8 | 5.0 | -10.0 |
| 9 | 0.0 | -3.0 |Number of samples
| 3.6 : 1 |
| --- |
| 3.0 : 1 |
| 1.9 : 1 |
| 1.7 : 1 |
| 1.5 : 1 |
| 1.0 : 1 |
| 1.0 : 1 |
| 0.7 : 1 |
| 0.5 : 1 |
Model score
F:M

## Slide 6
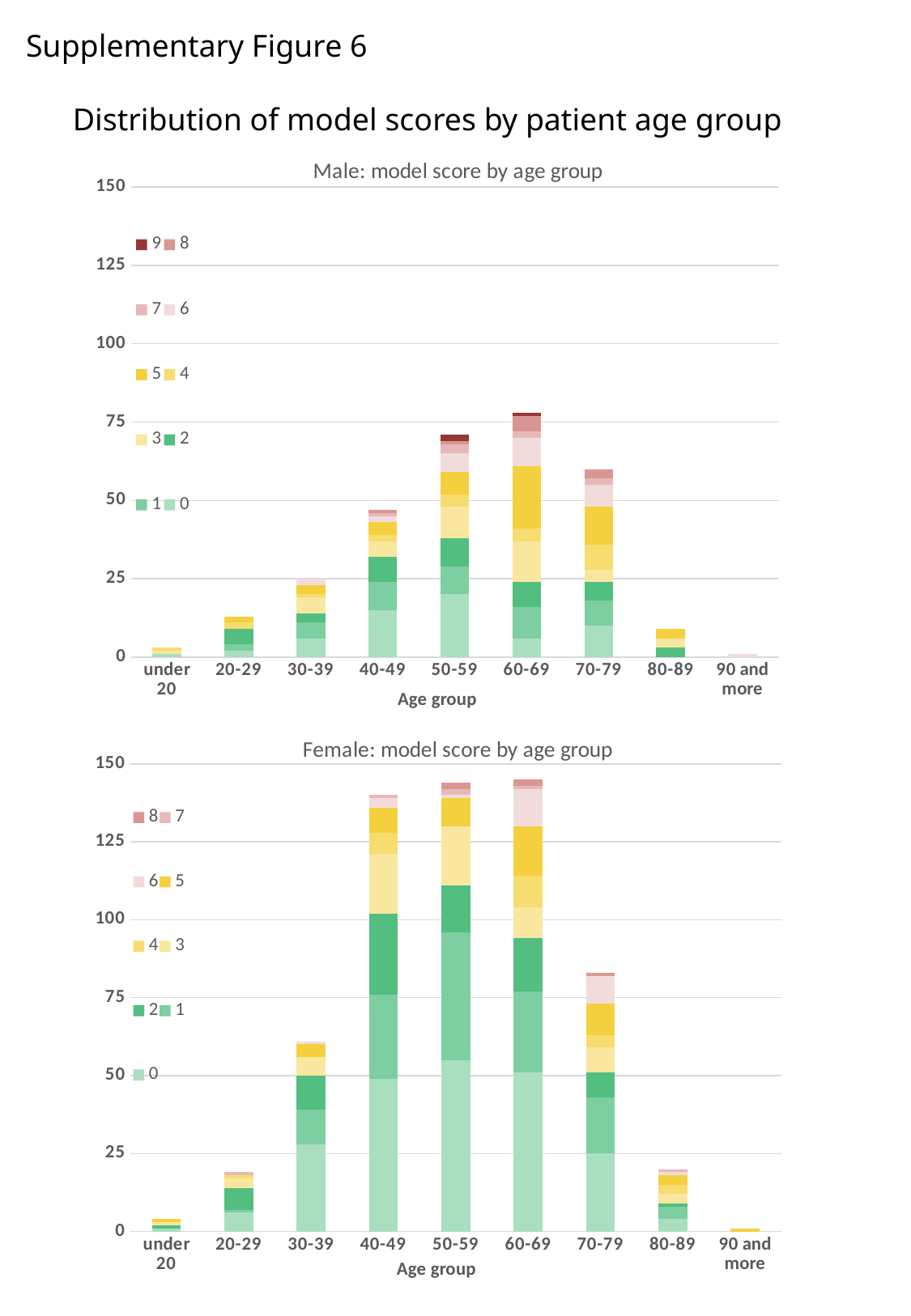

Supplementary Figure 6
Distribution of model scores by patient age group
### Chart: Male: model score by age group
| Category | 0 | 1 | 2 | 3 | 4 | 5 | 6 | 7 | 8 | 9 |
|---|---|---|---|---|---|---|---|---|---|---|
| under 20 | 1.0 | None | None | 1.0 | 1.0 | None | None | None | None | None |
| 20-29 | 2.0 | 2.0 | 5.0 | None | 2.0 | 2.0 | None | None | None | None |
| 30-39 | 6.0 | 5.0 | 3.0 | 5.0 | 1.0 | 3.0 | 2.0 | None | None | None |
| 40-49 | 15.0 | 9.0 | 8.0 | 5.0 | 2.0 | 4.0 | 2.0 | 1.0 | 1.0 | None |
| 50-59 | 20.0 | 9.0 | 9.0 | 10.0 | 4.0 | 7.0 | 6.0 | 3.0 | 1.0 | 2.0 |
| 60-69 | 6.0 | 10.0 | 8.0 | 13.0 | 4.0 | 20.0 | 9.0 | 2.0 | 5.0 | 1.0 |
| 70-79 | 10.0 | 8.0 | 6.0 | 4.0 | 8.0 | 12.0 | 7.0 | 2.0 | 3.0 | None |
| 80-89 | None | None | 3.0 | 3.0 | None | 3.0 | None | None | None | None |
| 90 and more | None | None | None | None | None | None | 1.0 | None | None | None |
### Chart: Female: model score by age group
| Category | 0 | 1 | 2 | 3 | 4 | 5 | 6 | 7 | 8 |
|---|---|---|---|---|---|---|---|---|---|
| under 20 | 1.0 | None | 1.0 | 1.0 | None | 1.0 | None | None | None |
| 20-29 | 6.0 | 1.0 | 7.0 | 3.0 | 1.0 | None | None | 1.0 | None |
| 30-39 | 28.0 | 11.0 | 11.0 | 6.0 | None | 4.0 | 1.0 | None | None |
| 40-49 | 49.0 | 27.0 | 26.0 | 19.0 | 7.0 | 8.0 | 3.0 | 1.0 | None |
| 50-59 | 55.0 | 41.0 | 15.0 | 19.0 | None | 9.0 | 1.0 | 2.0 | 2.0 |
| 60-69 | 51.0 | 26.0 | 17.0 | 10.0 | 10.0 | 16.0 | 12.0 | 1.0 | 2.0 |
| 70-79 | 25.0 | 18.0 | 8.0 | 8.0 | 4.0 | 10.0 | 9.0 | None | 1.0 |
| 80-89 | 4.0 | 4.0 | 1.0 | 3.0 | 3.0 | 3.0 | 1.0 | 1.0 | None |
| 90 and more | None | None | None | None | None | 1.0 | None | None | None |

## Slide 7
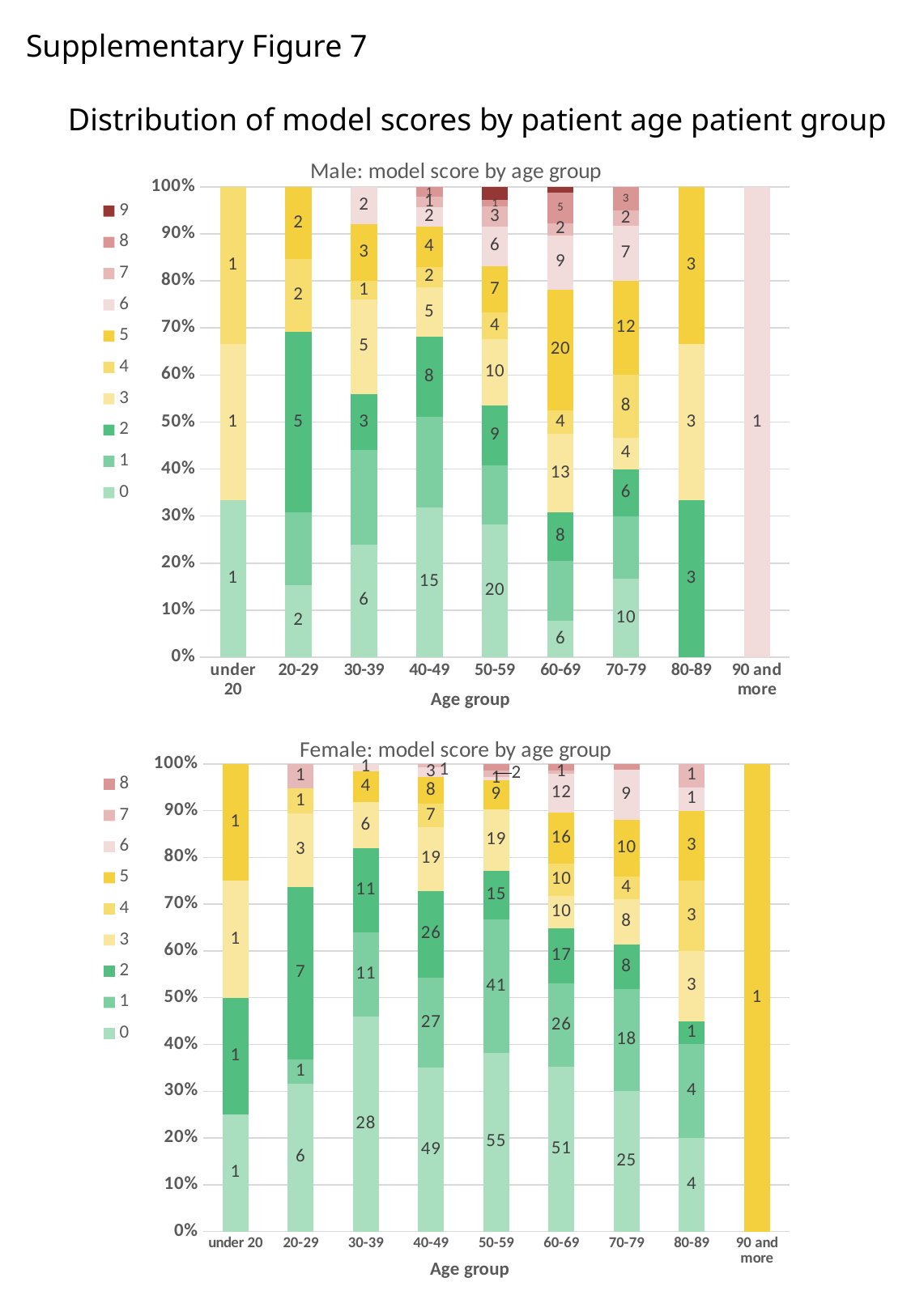

Supplementary Figure 7
Distribution of model scores by patient age patient group
### Chart: Male: model score by age group
| Category | 0 | 1 | 2 | 3 | 4 | 5 | 6 | 7 | 8 | 9 |
|---|---|---|---|---|---|---|---|---|---|---|
| under 20 | 1.0 | None | None | 1.0 | 1.0 | None | None | None | None | None |
| 20-29 | 2.0 | 2.0 | 5.0 | None | 2.0 | 2.0 | None | None | None | None |
| 30-39 | 6.0 | 5.0 | 3.0 | 5.0 | 1.0 | 3.0 | 2.0 | None | None | None |
| 40-49 | 15.0 | 9.0 | 8.0 | 5.0 | 2.0 | 4.0 | 2.0 | 1.0 | 1.0 | None |
| 50-59 | 20.0 | 9.0 | 9.0 | 10.0 | 4.0 | 7.0 | 6.0 | 3.0 | 1.0 | 2.0 |
| 60-69 | 6.0 | 10.0 | 8.0 | 13.0 | 4.0 | 20.0 | 9.0 | 2.0 | 5.0 | 1.0 |
| 70-79 | 10.0 | 8.0 | 6.0 | 4.0 | 8.0 | 12.0 | 7.0 | 2.0 | 3.0 | None |
| 80-89 | None | None | 3.0 | 3.0 | None | 3.0 | None | None | None | None |
| 90 and more | None | None | None | None | None | None | 1.0 | None | None | None |
### Chart: Female: model score by age group
| Category | 0 | 1 | 2 | 3 | 4 | 5 | 6 | 7 | 8 |
|---|---|---|---|---|---|---|---|---|---|
| under 20 | 1.0 | None | 1.0 | 1.0 | None | 1.0 | None | None | None |
| 20-29 | 6.0 | 1.0 | 7.0 | 3.0 | 1.0 | None | None | 1.0 | None |
| 30-39 | 28.0 | 11.0 | 11.0 | 6.0 | None | 4.0 | 1.0 | None | None |
| 40-49 | 49.0 | 27.0 | 26.0 | 19.0 | 7.0 | 8.0 | 3.0 | 1.0 | None |
| 50-59 | 55.0 | 41.0 | 15.0 | 19.0 | None | 9.0 | 1.0 | 2.0 | 2.0 |
| 60-69 | 51.0 | 26.0 | 17.0 | 10.0 | 10.0 | 16.0 | 12.0 | 1.0 | 2.0 |
| 70-79 | 25.0 | 18.0 | 8.0 | 8.0 | 4.0 | 10.0 | 9.0 | None | 1.0 |
| 80-89 | 4.0 | 4.0 | 1.0 | 3.0 | 3.0 | 3.0 | 1.0 | 1.0 | None |
| 90 and more | None | None | None | None | None | 1.0 | None | None | None |

## Slide 8
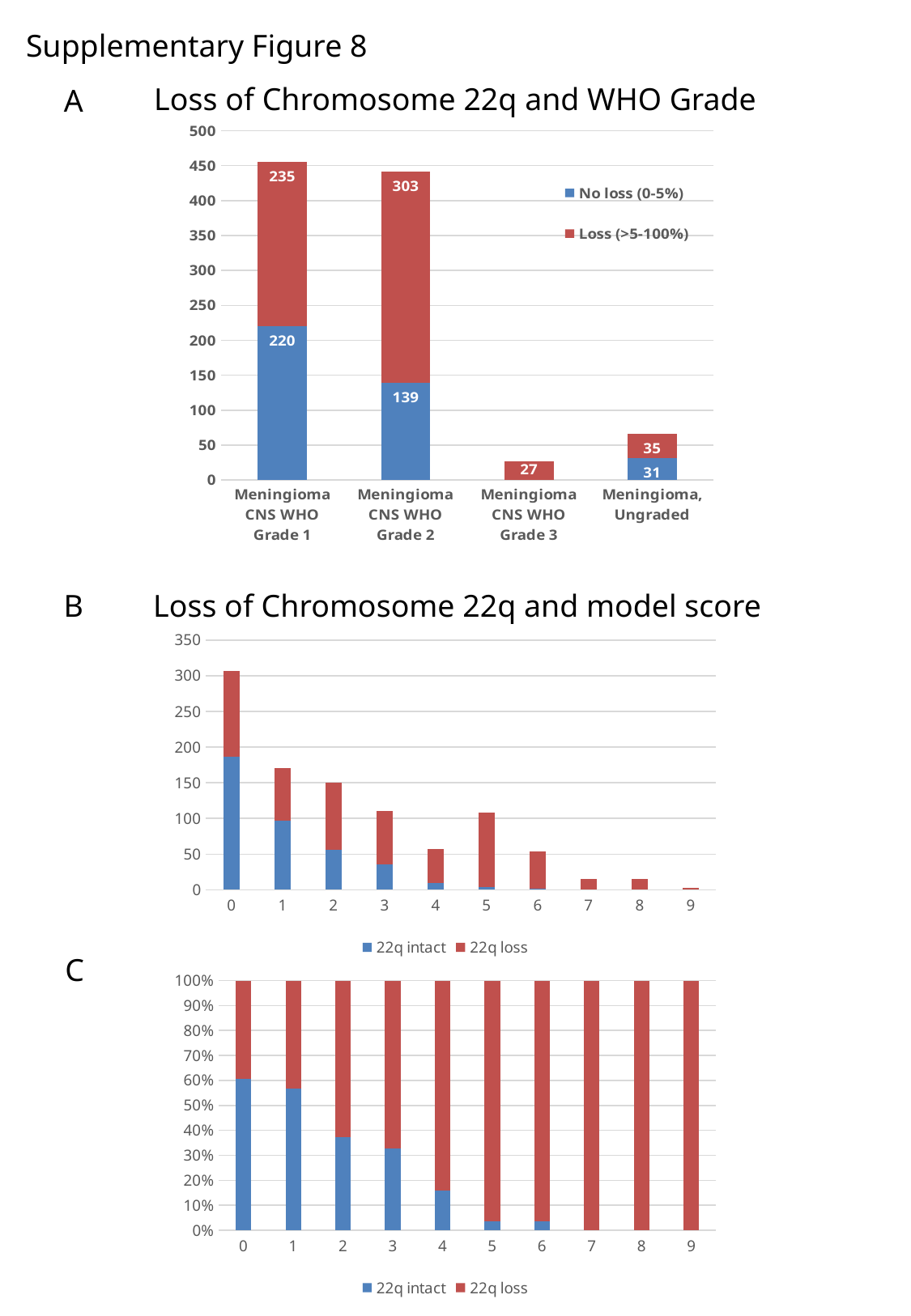

Supplementary Figure 8
Loss of Chromosome 22q and WHO Grade
A
### Chart
| Category | No loss (0-5%) | Loss (>5-100%) |
|---|---|---|
| Meningioma CNS WHO Grade 1 | 220.0 | 235.0 |
| Meningioma CNS WHO Grade 2 | 139.0 | 303.0 |
| Meningioma CNS WHO Grade 3 | None | 27.0 |
| Meningioma, Ungraded | 31.0 | 35.0 |B
Loss of Chromosome 22q and model score
### Chart
| Category | | |
|---|---|---|
| 0 | 186.0 | 121.0 |
| 1 | 97.0 | 74.0 |
| 2 | 56.0 | 94.0 |
| 3 | 36.0 | 74.0 |
| 4 | 9.0 | 48.0 |
| 5 | 4.0 | 104.0 |
| 6 | 2.0 | 52.0 |
| 7 | None | 15.0 |
| 8 | None | 15.0 |
| 9 | None | 3.0 |C
### Chart
| Category | | |
|---|---|---|
| 0 | 186.0 | 121.0 |
| 1 | 97.0 | 74.0 |
| 2 | 56.0 | 94.0 |
| 3 | 36.0 | 74.0 |
| 4 | 9.0 | 48.0 |
| 5 | 4.0 | 104.0 |
| 6 | 2.0 | 52.0 |
| 7 | None | 15.0 |
| 8 | None | 15.0 |
| 9 | None | 3.0 |
