# Supplementary material for: Testing Meningiomas With Methylation Arrays: Insights and Recommendations From a Large Single‐Centre Study
Source: Neuropathol Appl Neurobiol. 2025 May 13;51(3):e70018. doi: 10.1111/nan.70018 (PMC12070139; doi:10.1111/nan.70018)
Supplement: Supplementary file 2 — Table S1 Meningiomas diagnosed internally, with or without array testing. Table S2 Meningiomas referred for a second opinion, mostly for methylation array profiling. Table S3 Meningiomas with methylation profile, with a calibrated score of 0.9–1.0. Only these meningiomas were included in the subsequent analysis. Table S4 Meningiomas with methylation profile, with a calibrated score of 0.3 < 0.9. These meningiomas were excluded from the analysis. Table S5 Diagnosed grade and their allocation to the model scores of 0–9. Only samples with calibrated scores of 0.9 and higher were included in the analysis (n = 924), and meningiomas with no initial WHO grade (n = 66) were excluded. Table S6 Atypical meningiomas, CNS WHO Grade 2, their grading criterium (left column) and allocation to model score (0–9, top row). Table S7 Mitotic counts, grouped into strata (0–3, 4–7, 8–11, 12–15, 16–19 and 20–30) and allocation to the model scores, including CNS WHO Grade 1, 2 and 3 meningiomas. Table S8 CNS WHO Grade 2 meningiomas which were graded based on mitotic count only, i.e., excluding histology characteristics or brain invasion. Mitotic counts are grouped into strata (4–7, 8–11, 12–15, 16–20) and allocated to the model scores of 1–8. Table S9 Male‐to‐female ratios increase with higher model scores. Table S10 Chromosome 1p status in meningiomas of CNS WHO Grades 1, 2 and 3 with existing chromosome 22q deletion (> 5% of the chromosome) or intact chr 22q. For CNS WHO Grade 2 meningiomas, the data are separated by grading criteria (BI = brain invasion, Mit = mitotic counts, Histo = histological type, BI + Mit = brain invasion and increased mitotic count). See Figure 9 for the corresponding illustration. Table S11: Chromosome 22q status and initial WHO grade in 990 meningiomas with a calibrated score of 0.9 and higher. Table S12: Chromosome 22q status and model score, in 990 meningiomas with a calibrated score of 0.9 and higher. [file NAN-51-e70018-s001.docx]

### Supplementary tables

**Supplementary Table 1**: meningiomas diagnosed internally, with or without array testing.

| **Year of diagnosis** | **Grade 1** | | **Grade 2** | | **Grade 3** | | **No grade** | | **Total** |
| --- | --- | --- | --- | --- | --- | --- | --- | --- | --- |
|  | Array | No Array | Array | No Array | Array | No Array | Array | No Array |  |
| **2017** | 0 | 192 | 1 | 50 | 0 | 5 | 0 | 7 | **255** |
| **2018** | 2 | 190 | 3 | 32 | 0 | 1 | 3 | 5 | **236** |
| **2019** | 15 | 223 | 8 | 34 | 3 | 1 | 3 | 6 | **293** |
| **2020** | 25 | 206 | 37 | 22 | 2 | 2 | 2 | 5 | **301** |
| **2021** | 27 | 213 | 45 | 6 | 6 | 0 | 12 | 9 | **318** |
| **2022** | 71 | 191 | 61 | 4 | 7 | 2 | 12 | 2 | **350** |
| **2023** | 93 | 224 | 77 | 6 | 7 | 0 | 18 | 1 | **426** |
| Grand Total |  |  |  |  |  |  |  |  | **2179** |
| Σ Profiled | **233** |  | **232** |  | **25** |  | **50** |  | **540** |
| Σ Not profiled |  | **1439** |  | **154** |  | **11** |  | **35** | **1639** |
|  | Proportion of tumours profiled with methylation array | | | | | | | | |
| **2017** | 0.00 |  | 0.02 |  | 0.00 |  | 0.00 |  | 0.004 |
| **2018** | 0.01 |  | 0.09 |  | 0.00 |  | 0.38 |  | 0.034 |
| **2019** | 0.06 |  | 0.19 |  | 0.75 |  | 0.33 |  | 0.099 |
| **2020** | 0.11 |  | 0.63 |  | 0.50 |  | 0.29 |  | 0.219 |
| **2021** | 0.11 |  | 0.88 |  | 1.00 |  | 0.57 |  | 0.283 |
| **2022** | 0.27 |  | 0.94 |  | 0.78 |  | 0.86 |  | 0.431 |
| **2023** | 0.29 |  | 0.93 |  | 1.00 |  | 0.95 |  | 0.458 |
| 2017-2023 |  |  |  |  |  |  |  |  | **0.248** |

**Supplementary Table 2:** meningiomas referred for a second opinion, mostly for methylation array profiling.

| Year of diagnosis | Grade 1 | | Grade 2 | | Grade 3 | | No grade | | Total |
| --- | --- | --- | --- | --- | --- | --- | --- | --- | --- |
|  | Array | No Array | Array | No Array | Array | No Array | Array | No Array |  |
| 2017 | 0 | 2 | 0 | 0 | 0 | 0 | 0 | 0 | **2** |
| 2018 | 1 | 4 | 1 | 1 | 1 | 0 | 2 | 0 | **10** |
| 2019 | 14 | 3 | 5 | 0 | 2 | 0 | 12 | 1 | **37** |
| 2020 | 28 | 0 | 10 | 0 | 0 | 0 | 10 | 0 | **48** |
| 2021 | 39 | 0 | 24 | 0 | 3 | 0 | 24 | 2 | **92** |
| 2022 | 71 | 1 | 74 | 1 | 13 | 1 | 3 | 0 | **164** |
| 2023 | 128 | 4 | 191 | 0 | 15 | 0 | 15 | 0 | **353** |
| Grand Total |  |  |  |  |  |  |  |  | **706** |
| Σ Profiled | **281** |  | **305** |  | **34** |  | **66** |  | **686** |
| Σ Not profiled |  | **14** |  | **2** |  | **1** |  | **3** | **20** |
|  |  |  |  |  |  |  |  |  |  |

**Supplementary Table 3:** Meningiomas with methylation profile, with a calibrated score of 0.9-1.0. Only these meningiomas were included in the subsequent analysis.

| **Calibrated scores >=0.9** | **Ben** | **Int** | **SMARCE1** | **Mal** | **total** |
| --- | --- | --- | --- | --- | --- |
| Meningioma CNS WHO Grade 1 | 408 | 46 | 1 |  | **455** |
| Meningioma CNS WHO Grade 2 | 286 | 133 | 7 | 16 | **442** |
| Meningioma CNS WHO Grade 3 | 7 | 10 |  | 10 | **27** |
| Meningioma, Ungraded | 49 | 16 |  | 1 | **66** |
| Meningioma, all grades | **750** | **205** | **8** | **27** | **990** |
|  |  |  |  |  |  |
| Meningioma CNS WHO Grade 1 | 0.897 | 0.101 | 0.002 | 0.000 |  |
| Meningioma CNS WHO Grade 2 | 0.647 | 0.301 | 0.016 | 0.036 |  |
| Meningioma CNS WHO Grade 3 | 0.259 | 0.370 | 0.000 | 0.370 |  |
| Meningioma, Ungraded | 0.742 | 0.242 | 0.000 | 0.015 |  |
| Meningioma, all grades | **0.758** | **0.207** | **0.008** | **0.027** |  |

**Supplementary Table 4**: Meningiomas with methylation profile, with a calibrated score of 0.3-<0.9. These meningiomas were excluded from the analysis.

| **Calibrated scores 0.3 <0.9** | **Ben** | **Int** | **SMARCE1** | **Mal** | **total** |
| --- | --- | --- | --- | --- | --- |
| Meningioma CNS WHO Grade 1 | 53 | 15 | 0 | 4 | **72** |
| Meningioma CNS WHO Grade 2 | 60 | 51 | 1 | 12 | **124** |
| Meningioma CNS WHO Grade 3 | 8 | 8 | 0 | 7 | **23** |
| Meningioma, Ungraded | 13 | 3 | 0 | 1 | **17** |
| Meningioma, all grades | **135** | **77** | **0** | **23** | **236** |
|  |  |  |  |  |  |
| Meningioma CNS WHO Grade 1 | 0.736 | 0.208 | 0.014 | 0.042 |  |
| Meningioma CNS WHO Grade 2 | 0.484 | 0.411 | 0.008 | 0.097 |  |
| Meningioma CNS WHO Grade 3 | 0.375 | 0.333 | 0.000 | 0.292 |  |
| Meningioma, Ungraded | 0.765 | 0.176 | 0.000 | 0.059 |  |
| Meningioma, all grades | **0.570** | **0.325** | **0.000** | **0.097** |  |

**Supplementary Table 5**: Diagnosed grade and their allocation to the model scores 0-9. Only samples with calibrated scores of 0.9 and higher were included in the analysis (n=924), and meningiomas with no initial WHO grade (n=66) were excluded

|  | **Model score** | | | | | | | | | |  |
| --- | --- | --- | --- | --- | --- | --- | --- | --- | --- | --- | --- |
|  | **0** | **1** | **2** | **3** | **4** | **5** | **6** | **7** | **8** | **9** | Total |
| Meningioma CNS WHO Grade 1 | 279 | 0 | 128 | 4 | 29 | 15 | 0 | 0 | 0 | 0 | **455** |
| Meningioma CNS WHO Grade 2 | 0 | 171 | 0 | 106 | 14 | 85 | 50 | 8 | 8 | 0 | **442** |
| Meningioma CNS WHO Grade 3 | 0 | 0 | 0 | 0 | 4 | 3 | 4 | 6 | 7 | 3 | **27** |
| All grades | **279** | **171** | **128** | **110** | **47** | **103** | **54** | **14** | **15** | **3** | **924** |
|  | **Risk groups** | | | | | | | | | |  |
|  | 0, 1, 2 (low) | | | 3,4,5 (int) | | | 6,7,8,9 (high) | | | |  |
| Meningioma CNS WHO Grade 1 | n=407, 0.89 | | | n=48, 0.11 | | | n=0, 0.00 | | | |  |
| Meningioma CNS WHO Grade 2 | n=171, 0.39 | | | n=205, 0.46 | | | n=66, 0.15 | | | |  |
| Meningioma CNS WHO Grade 3 | n=0, 0.00 | | | n=7, 0.26 | | | n=20, 0.74 | | | |  |
| All grades | **0.63** | | | **0.29** | | | **0.13** | | | |  |

**Supplementary Table 6:** Atypical meningiomas, CNS WHO grade 2, their grading criterium (left column) and allocation to model score (0-9, top row).

| **Score**  **Criteria** | **0** | **1** | **2** | **3** | **4** | **5** | **6** | **7** | **8** | **9** | **Total** |
| --- | --- | --- | --- | --- | --- | --- | --- | --- | --- | --- | --- |
| Brain invasion | 0 | 55 | 0 | 13 | 1 | 16 | 8 | 0 | 2 | 0 | 95 |
| Mitotic count | 0 | 98 | 0 | 52 | 6 | 51 | 29 | 7 | 5 | 0 | 248 |
| Histology type | 0 | 16 | 0 | 37 | 6 | 8 | 3 | 0 | 0 | 0 | 70 |
| Brain invasion + mitoses | 0 | 2 | 0 | 3 | 1 | 10 | 10 | 1 | 1 | 0 | 28 |
| **Total model score** | **0** | **171** | **0** | **105** | **14** | **85** | **50** | **8** | **8** | **0** | **441** |
|  |  |  |  |  |  |  |  |  |  |  |  |
| Brain invasion | 0.00 | 0.58 | 0.00 | 0.14 | 0.01 | 0.17 | 0.08 | 0.00 | 0.02 | 0.00 | **0.22** |
| Mitotic count | 0.00 | 0.40 | 0.00 | 0.21 | 0.02 | 0.21 | 0.12 | 0.03 | 0.02 | 0.00 | **0.56** |
| Histology type | 0.00 | 0.23 | 0.00 | 0.53 | 0.09 | 0.11 | 0.04 | 0.00 | 0.00 | 0.00 | **0.16** |
| Brain invasion + mitoses | 0.00 | 0.07 | 0.00 | 0.11 | 0.04 | 0.36 | 0.36 | 0.04 | 0.04 | 0.00 | **0.06** |
| **Proportion of total (441)** | **0.00** | **0.39** | **0.00** | **0.24** | **0.03** | **0.19** | **0.11** | **0.02** | **0.02** | **0.00** | **1.00** |

**Supplementary Table 7:** Mitotic counts, grouped into strata (0-3, 4-7, 8-11, 12-15, 16-19, and 20-30) and allocation to the model scores, including CNS WHO Grade 1, 2, and 3 meningiomas

| **Mitoses**  **Model Score** | | **0-3** | | **4-7** | | **8-11** | | **12-15** | **16-19** | | **20-30** | **Score total** |  |
| --- | --- | --- | --- | --- | --- | --- | --- | --- | --- | --- | --- | --- | --- |
| **0** | | 157 | |  | |  | |  |  | |  | **157** |  |
| **1** | | 63 | | 98 | | 5 | |  |  | |  | **166** |  |
| **2** | | 78 | | 1 | |  | |  |  | |  | **79** |  |
| **3** | | 38 | | 46 | | 9 | | 2 | 1 | |  | **96** |  |
| **4** | | 24 | | 5 | | 2 | |  |  | | 2 | **33** |  |
| **5** | | 26 | | 51 | | 9 | | 2 | 5 | | 1 | **94** |  |
| **6** | | 7 | | 28 | | 8 | | 5 | 2 | | 4 | **54** |  |
| **7** | |  | | 3 | | 3 | | 3 |  | | 5 | **14** |  |
| **8** | | 1 | | 2 | | 2 | | 1 | 4 | | 5 | **15** |  |
| **9** | |  | |  | |  | | 1 | 1 | | 1 | **3** |  |
| **Group total** | | **394** | | **234** | | **38** | | **14** | **13** | | **18** | **711** |  |
|  |  | |  | |  | |  | |  |  | |  | |
| **Mitoses**  **Risk group** | **0-3** | | **4-7** | | **8-11** | | **12-15** | | **16-19** | **20-30** | |  | |
| Low (0,1,2) | 0.76 | | 0.42 | | 0.13 | | 0.00 | | 0.00 | 0.00 | |  | |
| Intermediate (3,4,5) | 0.22 | | 0.44 | | 0.53 | | 0.29 | | 0.46 | 0.17 | |  | |
| High (6,7,8,9) | 0.02 | | 0.14 | | 0.34 | | 0.71 | | 0.54 | 0.83 | |  | |

**Supplementary Table 8**: CNS WHO Grade 2 meningiomas which were graded based on mitotic count only, i.e. excluding histology characteristics or brain invasion. Mitotic counts are grouped into strata (4-7, 8-11, 12-15, 16-20) and allocated to the model scores 1-8.

| **Mitoses**  **Model score** | **4-7** | **8-11** | **12-15** | **16-19** | Score Total |  |
| --- | --- | --- | --- | --- | --- | --- |
| **1** | 93 | 5 |  |  | **98** |  |
| **3** | 41 | 8 | 2 | 1 | **52** |  |
| **4** | 5 | 1 |  |  | **6** |  |
| **5** | 38 | 6 | 2 | 4 | **50** |  |
| **6** | 19 | 6 | 2 | 2 | **29** |  |
| **7** | 3 | 1 | 3 |  | **7** |  |
| **8** |  | 2 | 1 | 2 | **5** |  |
| **Group total** | **199** | **29** | **10** | **9** | **247** |  |
| **Model Score** | **Proportion within score** | | | |  |  |
| **1** | 0.95 | 0.05 | 0.00 | 0.00 | **1.00** |  |
| **3** | 0.79 | 0.15 | 0.04 | 0.02 | **1.00** |  |
| **4** | 0.83 | 0.17 | 0.00 | 0.00 | **1.00** |  |
| **5** | 0.76 | 0.12 | 0.04 | 0.08 | **1.00** |  |
| **6** | 0.66 | 0.21 | 0.07 | 0.07 | **1.00** |  |
| **7** | 0.43 | 0.14 | 0.43 | 0.00 | **1.00** |  |
| **8** | 0.00 | 0.40 | 0.20 | 0.40 | **1.00** |  |
| **Model Score** | **Proportion within mitotic range** | | | |  |  |
| **1** | 0.47 | 0.17 | 0.00 | 0.00 |  |  |
| **3** | 0.21 | 0.28 | 0.20 | 0.11 |  |  |
| **4** | 0.03 | 0.03 | 0.00 | 0.00 |  |  |
| **5** | 0.19 | 0.21 | 0.20 | 0.44 |  |  |
| **6** | 0.10 | 0.21 | 0.20 | 0.22 |  |  |
| **7** | 0.02 | 0.03 | 0.30 | 0.00 |  |  |
| **8** | 0.00 | 0.07 | 0.10 | 0.22 |  |  |
|  | **1.00** | **1.00** | **1.00** | **1.00** |  |  |

**Supplementary Table 9:** Male-to-female ratios increase with higher model scores.

| **Patient sex**  **Model score** | **F** | **M** |  | **F** | **M** |
| --- | --- | --- | --- | --- | --- |
| **0** | 240 | 67 |  | 0.78 | 0.22 |
| **1** | 128 | 43 |  | 0.75 | 0.25 |
| **2** | 99 | 51 |  | 0.66 | 0.34 |
| **3** | 69 | 41 |  | 0.63 | 0.37 |
| **4** | 34 | 23 |  | 0.60 | 0.40 |
| **5** | 54 | 54 |  | 0.50 | 0.50 |
| **6** | 27 | 27 |  | 0.50 | 0.50 |
| **7** | 6 | 9 |  | 0.40 | 0.60 |
| **8** | 5 | 10 |  | 0.33 | 0.67 |
| **9** | 0 | 3 |  | 0.00 | 1.00 |
| **Total** | **662** | **328** |  |  |  |

**Supplementary Table 10**: Chromosome 1p status in meningiomas of CNS WHO grade 1, 2, and 3 with existing chromosome 22q deletion (>5% of the chromosome)), or intact chr 22q. For CNS WHO grade 2 meningiomas, the data are separated by grading criteria (BI= brain invasion, Mit= mitotic counts, Histo= histological type, BI+Mit = brain invasion and increased mitotic count. See Figure 9 for the corresponding illustration.

|  | BI | Mit | Histo | BI+Mit | **Sum** |
| --- | --- | --- | --- | --- | --- |
| **Chr 1p intact, Chr 22 intact** |  |  |  |  |  |
| Meningioma CNS WHO Grade 1 |  |  |  |  | 184 |
| Meningioma CNS WHO Grade 2 | 45 | 37 | 17 | 2 | 101 |
| Meningioma, Ungraded |  |  |  |  | 21 |
| Subtotal |  |  |  |  | **306** |
|  |  |  |  |  |  |
| **Chr 1p intact, Chr 22 deleted** |  |  |  |  |  |
| Meningioma CNS WHO Grade 1 |  |  |  |  | 129 |
| Meningioma CNS WHO Grade 2 | 13 | 75 | 4 | 2 | 94 |
| Meningioma, Ungraded |  |  |  |  | 11 |
| Subtotal |  |  |  |  | **234** |
| **Chr 1p deleted, Chr 22 intact** |  |  |  |  |  |
| Meningioma CNS WHO Grade 1 |  |  |  |  | **36** |
| Meningioma CNS WHO Grade 2 | 5 | 14 | 17 | 2 | **38** |
| Meningioma, Ungraded |  |  |  |  | **10** |
| Subtotal |  |  |  |  | **84** |
|  |  |  |  |  |  |
| **Chr 1p deleted, Chr 22 deleted** |  |  |  |  |  |
| Meningioma CNS WHO Grade 1 |  |  |  |  | 106 |
| Meningioma CNS WHO Grade 2 | 32 | 122 | 32 | 22 | 208 |
| Meningioma CNS WHO Grade 3 |  |  |  |  | 27 |
| Meningioma, Ungraded |  |  |  |  | 24 |
| Subtotal |  |  |  |  | **365** |
|  |  |  |  |  |  |

**Supplementary Table 11**: Chromosome 22q status and initial WHO grade in 990 meningiomas with a calibrated score of 0.9 and higher

| **Chr 22 Status**  **Grade** | **22q intact** | **22q loss** | **Total** |  | **22q intact** | **22q loss** |
| --- | --- | --- | --- | --- | --- | --- |
| **Meningioma CNS WHO Grade 1** | 220 | 235 | 455 |  | 0.48 | 0.52 |
| **Meningioma CNS WHO Grade 2** | 139 | 303 | 442 |  | 0.31 | 0.69 |
| **Meningioma CNS WHO Grade 3** | 0 | 27 | 27 |  | 0.00 | 1.00 |
| **Meningioma, Ungraded** | 31 | 35 | 66 |  | 0.47 | 0.53 |
| **All meningiomas** | **390** | **600** | **990** |  | **0.39** | **0.61** |

**Supplementary Table 12**: Chromosome 22q status and model score, in 990 meningiomas with a calibrated score of 0.9 and higher

| **Chr 22 status**  **Model score** | **22q intact** | **22q loss** | **Total** |  | **22q intact** | **22q loss** |
| --- | --- | --- | --- | --- | --- | --- |
| 0 | 186 | 121 | 307 |  | 0.61 | 0.39 |
| 1 | 97 | 74 | 171 |  | 0.57 | 0.43 |
| 2 | 56 | 94 | 150 |  | 0.37 | 0.63 |
| 3 | 36 | 74 | 110 |  | 0.33 | 0.67 |
| 4 | 9 | 48 | 57 |  | 0.16 | 0.84 |
| 5 | 4 | 104 | 108 |  | 0.04 | 0.96 |
| 6 | 2 | 52 | 54 |  | 0.04 | 0.96 |
| 7 | 0 | 15 | 15 |  | 0.00 | 1.00 |
| 8 | 0 | 15 | 15 |  | 0.00 | 1.00 |
| 9 | 0 | 3 | 3 |  | 0.00 | 1.00 |

## Supplementary figure legends

**Supplementary Figure 1**: Proportion of meningiomas on which methylation array profiling and risk prediction were performed, direct referrals. Due to the nature of the request, nearly all referred tumours underwent methylation profiling. The corresponding data is in Table 2.

**Supplementary figure 2:** meningiomas grade and allocation to MCs (all profiled tumours with a calibrated score of 0.3 up to 0.9. The allocation of the WHO grades to the MCs benign, intermediate, and malignant is similar to those with a calibrated score of 0.9 and above. Corresponding data in Table 4.

**Supplementary figure 3**: granular analysis and allocation of the mitotic counts to the model scores 0-9. Upper graph, all mitotic counts, i.e. of meningiomas grade 1, 2, and 3. Lower graph, allocation of grade 2 meningiomas only, showing an allocation of higher mitotic counts towards higher model scores. Corresponding data in table 9 (upper graph) and 10 (lower graph).

**Supplementary figure 4:** model score and corresponding mitotic counts of all meningiomas. The upper graph **(A)** shows the absolute frequency and the centre graph **(B)** the relative frequency. See also **Table 9** for corresponding data. **C,** Mitotic count and CDKN2A/B status. X axis, mitotic count (expressed in mitoses / 10 HPF), Y axis CDKN2A/B value determined from the copy number plot. There is a weak correlation between mitotic count and CDKN1A/B status, with a Pearson Coefficient of 0.38.

**Supplementary Figure 5**: upper graph: distribution of patients' sex and grade of meningiomas. There is a preponderance of female sex (2.4:1) in grade one meningiomas, a reduction of female representation in grade 2 meningiomas (1.6:1), and a male predominance in grade 3 meningiomas (0.7:1). Lower graph: in keeping with the decreasing female dominance with increasing grade, the same trend can be observed when plotting the sex distribution against the model score, showing an even stronger predominance of female sex in score 0 tumours (3.6:1) and a stronger prevalence of male sex in higher scores, i.e. 0.7:1 in score 7 and 0.5:1in score 8 tumours. See supplementary table 1 for corresponding data

**Supplementary Figure 6:** Distribution of model scores across patient age ranges (absolute numbers); upper graph male patients, lower graph female patients. In keeping with the more frequent occurrence of high-grade meningioma in males, this graph shows that the high-risk groups are more represented in male patients. It also shows that meningiomas operated in young females do not have a higher proportion of intermediate and high-risk scores than in the middle-aged and elderly population.

**Supplementary Figure 7**: Distribution of model scores across patient age ranges (as a fraction of 1 for each stratum); upper graph male patients, lower graph female patients. In keeping with the more frequent occurrence of high-grade meningioma in males, this graph shows that the high-risk groups are more represented in male patients. It also shows that meningiomas operated in young females do not have a higher proportion of intermediate and high-risk scores than in the middle-aged and elderly population.

**Supplementary figure 8:** A, loss of chromosome 22q is seen in approximately 50% of grade 1 tumours and this proportion increases in grade 2 tumours whilst all grade 3 tumours have a loss of chromosome 22q. The corresponding data is in supplementary table 2. B, C: model score and corresponding mitotic counts of all meningiomas. The upper graph shows the absolute frequency and the lower graph the relative frequency. See also Table 9 for corresponding data.
